# Retinal Changes From Hyperopia to Myopia: Not All Diopters Are Created Equal

**DOI:** 10.1167/iovs.65.5.25

**Published:** 2024-05-17

**Authors:** Fabian Yii, Miguel O. Bernabeu, Baljean Dhillon, Niall Strang, Tom MacGillivray

**Affiliations:** 1 Centre for Clinical Brain Sciences, University of Edinburgh, Edinburgh, United Kingdom; 2 Curle Ophthalmology Laboratory, Institute for Regeneration and Repair, University of Edinburgh, Edinburgh, United Kingdom; 3 Centre for Medical Informatics, Usher Institute, University of Edinburgh, Edinburgh, United Kingdom; 4 The Bayes Centre, University of Edinburgh, Edinburgh, United Kingdom; 5 Princess Alexandra Eye Pavilion, Edinburgh, United Kingdom; 6 Department of Vision Sciences, Glasgow Caledonian University, Glasgow, United Kingdom

**Keywords:** myopia, hyperopia, refractive error, retinal vasculature, optic disc, fovea

## Abstract

**Purpose:**

To quantitatively characterize retinal changes across different quantiles of refractive error in 34,414 normal eyes of 23,064 healthy adults in the UK Biobank.

**Methods:**

Twelve optic disc (OD), foveal and vascular parameters were derived from color fundus photographs, correcting for ocular magnification as appropriate. Quantile regression was used to test the independent associations between these parameters and spherical equivalent refraction (SER) across 34 refractive quantiles (high hyperopia to high myopia)—controlling for age, sex and corneal radius.

**Results:**

More negative SER was *nonlinearly* associated with greater Euclidian (largely horizontal) OD-fovea distance, larger OD, less circular OD, more obliquely orientated OD (superior pole tilted towards the fovea), brighter fovea, lower vascular complexity, less tortuous vessels, more concave (straightened out towards the fovea) papillomacular arterial/venous arcade and wider central retinal arterioles/venules. In myopia, these parameters varied more strongly with SER as myopia increased. For example, while every standard deviation (SD) decrease in vascular complexity was associated with 0.63 D (right eye: 95% confidence interval [CI], 0.58–0.68) to 0.68 D (left eye: 95% CI, 0.63–0.73) higher myopia in the quantile corresponding to −0.60 D, it was associated with 1.61 D (right eye: 95% CI, 1.40–1.82) to 1.70 D (left eye: 95% CI, 1.56–1.84) higher myopia in the most myopic quantile. OD-fovea angle (degree of vertical separation between OD and fovea) was found to vary linearly with SER, but the magnitude was of little practical importance (less than 0.10 D variation per SD change in angle in almost all refractive quantiles) compared with the changes in OD-fovea distance.

**Conclusions:**

Several interrelated retinal changes indicative of an increasing (nonconstant) rate of mechanical stretching are evident at the posterior pole as myopia increases. These changes also suggest that the posterior pole stretches predominantly in the temporal horizontal direction.

That myopic fundi exhibit characteristic changes has been noted since Helmholtz's epochal invention of the ophthalmoscope.^1^^,^^2^ Evidence recently reviewed by Jonas et al.^3^ strongly suggests that the retro-equatorial region of the globe is the centre of myopic ocular expansion, tying in with observations that myopic changes are mostly confined to the posterior half of the eye. These changes are generally ascribed to the biomechanical stretching of posterior ocular tissues and may, therefore, be conceived as the mechanistic basis for various sight-threatening sequelae of myopia—notably pathologic myopia or myopic maculopathy, a disease that primarily affects the posterior pole.^4^

Motivated in part by the established association between myopia and glaucoma,^5^ several studies have looked at the influence of myopia on optic nerve head parameters such as the optic disc (OD) area and OD tilt using fundus imaging.^6^^–^^11^ Vascular parameters, including arteriolar or venular calibre, tortuosity and fractal dimension, have also been explored by studies interested in elucidating the confounding effect of refractive error on the measurements of vascular geometry or studies directly interested in inferring the effect of refractive error on ocular blood flow.^12^^–^^21^ Despite these efforts, much remains to be learned about the nature of associations between these parameters and refractive error, not least because previous studies almost invariably considered different parameters in silos and with different diseases (e.g., diabetic retinopathy) or purposes in mind. The relative (adjusted) variation in different parameters across the refractive error spectrum, for example, remains unknown. Besides, whether the strength and direction of these associations depend on the subtype and severity of refractive error remains an open question. One may hypothesize that most of these associations, if present, are likely to vary across the refractive error spectrum in a much more complex way than previously implicitly assumed (nonvarying effects), considering that the risk of myopic complications such as myopic maculopathy and retinal detachment is known to increase in a highly nonlinear fashion with increasing myopia.^22^

Furthermore, parameters pertaining to the relative position of fundus landmarks remain understudied, such as the distance and angle between the OD and fovea.^23^^,^^24^ The influence of myopia on the course of the papillomacular vascular arcade has also not been quantitatively and robustly analyzed. The angle kappa computed by previous work^25^^,^^26^ has limitations because it fails to capture the overall parabolic course of the vascular arcade and thus cannot differentiate between arcades with comparable vertical displacement (i.e., separation between superior and inferior vessels) along the fovea but different radii of curvature near the optic nerve head. These topographical parameters merit further investigation because *collectively* (considered together and controlling for one another), they may provide insights into the nature of myopic retinal stretching (isotropic versus anisotropic) at the posterior pole. In addition, the brightness of the foveal region may be related to refractive error, considering that findings from recent studies using deep learning (DL) to predict refractive error^27^ and future development of high myopia^28^ from fundus photographs consistently highlighted the fovea as an important region of interest.

It is also worth highlighting that very little attention has hitherto been given to hyperopia. Considering that hyperopia, like myopia,^29^ is predominantly (though not exclusively) axial in nature but represents the other end of the axial length (AL) spectrum,^30^ exploring retinal changes in this subgroup is also useful because it allows for comparative investigation of retinal changes with and without myopia. A thorough characterisation of retinal alterations across refractive error could prove valuable in improving our understanding of the pathophysiology of myopia and its structural sequalae, which may in turn facilitate personalized prediction of myopic complications. In light of this, we aimed to characterize changes in a wide range of OD, foveal and retinal vascular parameters across different levels of refractive error using a flexible regression technique in a large cohort of healthy UK-based adults.

## Methods

### Selection of Participants

The UK Biobank is a large-scale biomedical database with richly phenotyped data from half a million residents in the United Kingdom. As the UK Biobank has prior Research Tissue Bank approval from the North West Multi-Centre Research Ethics Committee (06/MRE08/65), a separate ethical clearance was not required for the present study.

**Figure 1.**
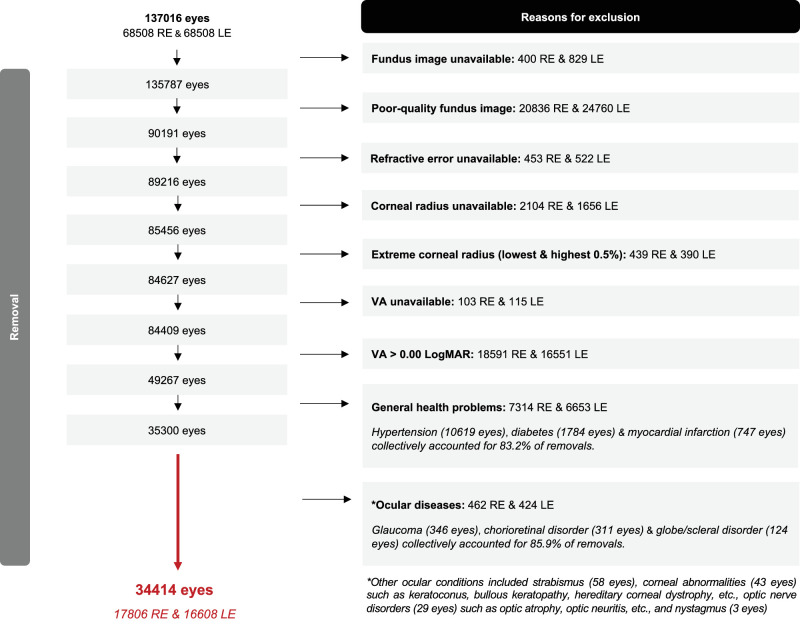
Flow diagram detailing each step of the participant selection process.

A total of 68,508 phakic participants in the UK Biobank underwent a standardized ophthalmic assessment.^31^ The flow diagram in Figure 1 details how the participants included in the present study were selected. Briefly, eyes with fundus photographs of “reject” quality (e.g., severe underexposure) as determined automatically using a validated DL model^32^ were removed to ensure only images suitable for subsequent automated analysis were included. Eyes with missing refractive error, keratometry or distance visual acuity (VA) were excluded. We removed eyes with extreme (top and bottom 0.5% of the distribution) corneal radius of curvature (CR) to minimize the influence of refractive ametropia on the associations between retinal parameters and refractive error. Eyes with poor VA (worse than 0.00 logMAR) were further removed. Both eyes of participants with systemic or health condition(s) as identified by linked health care data (biobank.ndph.ox.ac.uk/ukb/ukb/docs/first_occurrences_outcomes.pdf) were excluded, with the majority (83.1%) removed for having a history of hypertension, diabetes and/or myocardial infarction. Eyes with corneal abnormalities including keratoconus and posterior ocular conditions (also identified using linked health care data) were further removed, with 85.9% of all removals attributable to glaucoma, chorioretinal disorder or globe/scleral disorder (e.g., degenerative myopia, posterior staphyloma). A total of 34,414 normal eyes of 23,064 healthy participants were subsequently analyzed.

### Instrumentation

Refractive error and keratometry were measured automatically without cycloplegia using a Tomey RC-5000 Autorefractometer (Tomey, Nagoya, Japan). Spherical equivalent refraction (SER) was defined as spherical power + 0.5 × cylindrical power, while CR was given by the mean radius of the steepest and flattest corneal meridians. VA was measured using a logMAR chart (Precision Vision, LaSalle, IL, USA) on a digital screen at 4 m or 1 m (if a participant was unable to read at 4 m, in which case they would have been excluded from the study due to poor VA), with habitual distance correction in place. The test would terminate when two or more letters were read incorrectly. Macula-centred fundus photographs (45-degree field of view; 2048 × 1536 pixels) were captured using a digital Topcon-1000 integrated ophthalmic camera (Topcon 3D OCT1000 Mark II; Topcon Corp., Tokyo, Japan). Goldmann-correlated IOP was measured using the Ocular Response Analyzer (Reichert Corp., Philadelphia, PA, USA). The right eye was always measured before the left eye.

### Retinal Parameters

**Figure 2.**
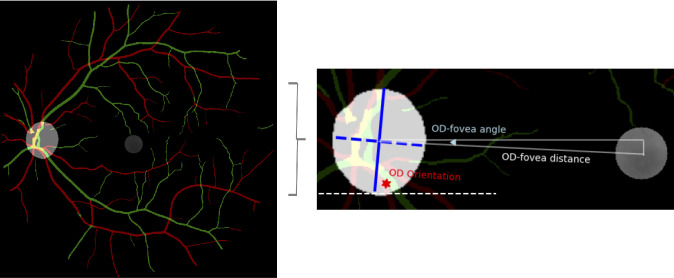
*Left*: OD, fovea, artery (*red*) and vein (*green*) segmentation masks. Central retinal arteriolar/venular equivalent, papillomacular arterial/venous concavity as well as vessel (i.e., artery and vein segmented simultaneously) tortuosity and fractal dimension were computed based on the segmented vasculature. *Right*: OD and foveal parameters, including OD-fovea angle, OD orientation, OD-fovea distance, OD ovality (i.e., OD major axis length, represented by the *blue solid line*, divided by OD minor axis length, represented by the *blue dotted line*), OD area (OD major axis length × OD minor axis length × Π / 4) and foveal pixel intensity (i.e., median pixel intensity of the foveal mask on the right, adjusted for the background fundus intensity), were derived from the OD and fovea segmentation masks.

Twelve retinal parameters, including OD orientation, OD ovality, OD area, OD-fovea distance, OD-fovea angle, foveal pixel intensity (FPI), central retinal arteriolar equivalent (CRAE), central retinal venular equivalent (CRVE), papillomacular arterial concavity, papillomacular venous concavity, vessel tortuosity and vessel fractal dimension (FD), were derived (Fig. 2). To do this, regions of interest were first automatically and semantically segmented, that is, pixel-by-pixel segmentation not based on a predefined shape, using validated DL models. OD was segmented using a MobileNetV3-Large model previously trained on 299 fundus photographs in the UK Biobank, which was shown to outperform other DL models and achieved a Dice score of 95.3% on the unseen test set.^33^ Retinal artery and vein were segmented using AutoMorph.^34^ The fovea was segmented using a DU-Net model adapted from Wang et al.^35^ by VAMPIRE (vampire.computing.dundee.ac.uk) and was postprocessed to ensure all segmented foveae had a similar size (1% of the fundus) and shape (circular).

The *regionprops* function in MATLAB (MathWorks, Natick, MA, USA) was used to compute OD orientation, OD major axis length and OD minor axis length. OD orientation referred to the angle between the horizontal axis of the fundus photograph and the major axis of the OD, ranging from −90 to 90 degrees, where a more negative (positive) value meant that the disc appeared less vertically orientated with its superior pole tilting towards the fovea in the right (left) eye. OD ovality was given by the ratio of the major axis length to the minor axis length. A larger value meant that the disc was stretched along the major axis and took on a less circular or more oval appearance en face. OD area was computed using the standard area formula for an ellipse (major axis length × minor axis length × Π / 4). OD-fovea distance referred to the shortest (Euclidian) distance between the OD centroid and foveal centroid, primarily reflecting the extent of *horizontal* OD-fovea separation. In keeping with previous studies,^24^^,^^36^ OD-fovea angle was defined as the angle between the horizontal line through the OD centroid and the line connecting the OD centroid to the foveal centroid, which mainly reflected the degree of *vertical* OD-fovea separation. The angle was computed using the following equation:

(1) $$\begin{array}{c} tan^{-1}\left(\frac{OD_{y}-\;macula_{y}}{abs\left(OD_{x}-\;macula_{x}\right)}\right)\; \end{array}$$

where subscripts ***x*** and ***y*** denote the x and y coordinates of the OD or macular centroid, while ***tan^−^^1^*** represents the inverse tangent function. We used absolute difference (***abs***) in the denominator (horizontal distance between OD and foveal centroids) so that a more negative value indicated that the OD was sitting higher than the fovea irrespective of whether it was right or left eye. FPI represented the median pixel intensity (grayscale) of the fovea, adjusted for the median background intensity of the whole fundus to account for differences in background illumination. A more negative FPI value meant that the fovea appeared brighter.

**Figure 3.**
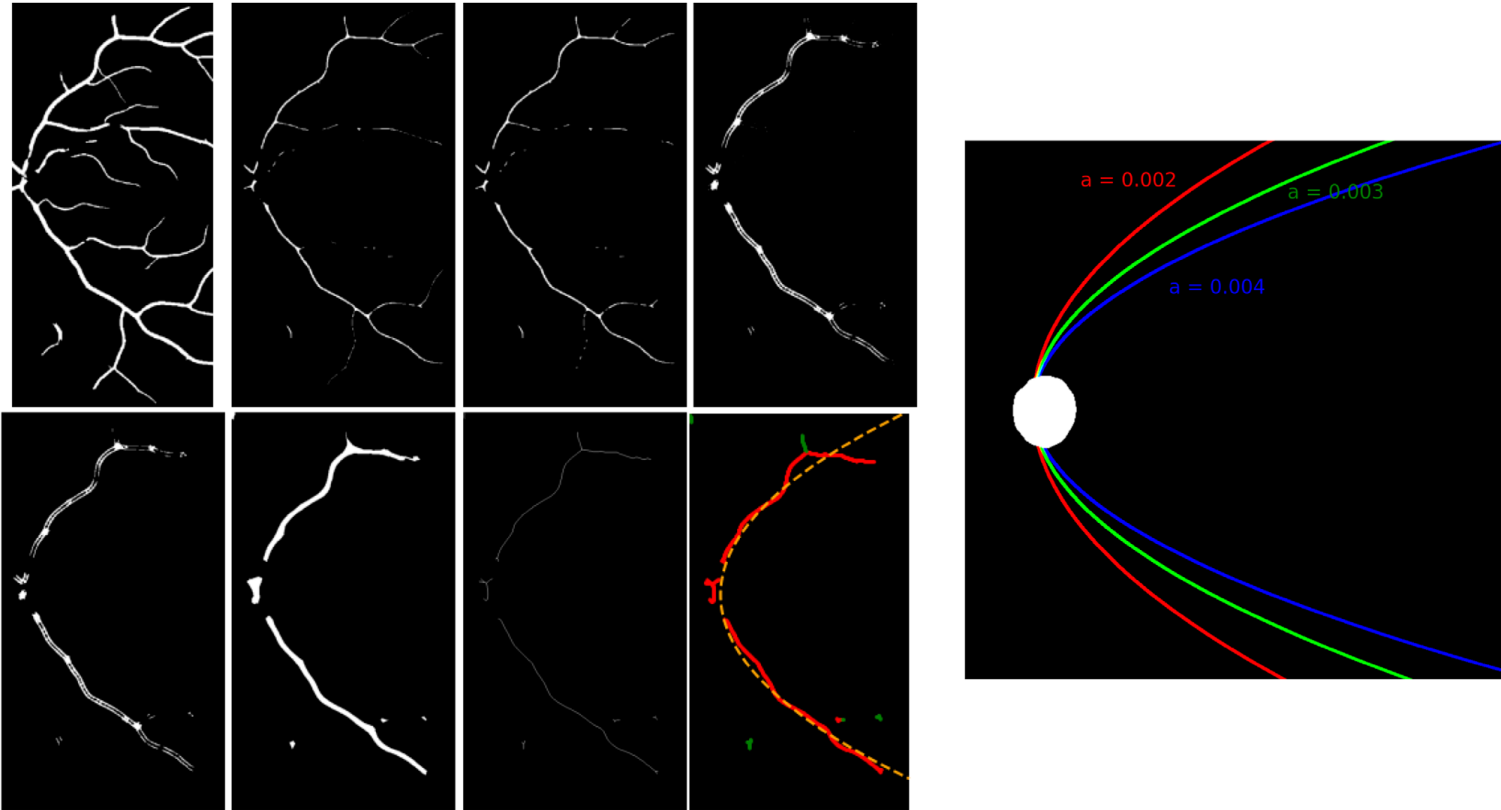
*Left*: Pipeline designed to extract the papillomacular arterial or venous arcade (artery shown in this example) based on which the degree of vascular concavity is estimated (proceeds horizontally from *top left* to *bottom right*). The segmentation mask is first cropped from 912 by 912 pixels to 912 by 450 pixels (*top left*). The mask is then padded and distance transformed (second image), followed by morphological area opening (third image), to remove the small vessels. Circle Hough transform is then used to detect and extract the main vascular arcade (fourth image). After this, morphological area opening (fifth image) is applied again to attenuate/remove isolated vascular segments inadvertently magnified by the circle Hough transform. This is followed by morphological closing (sixth image) to close the gaps in the vascular arcade and then skeletonisation (reduce vascular arcade to 1 pixel wide) to facilitate model fitting (seventh image). Finally, a parabola (quadratic function; *a* × *x*^2^ + *B* × *x* + *c*) is fitted to the skeletonized vessel (eighth image) using RANSAC, an iterative algorithm noted for its robustness to outliers (*green pixels* are identified as outliers, which have no influence on the fitting process). *Right*: The absolute value of parameter *a* describes how concave the vascular arcade is, where a larger value indicates that it is more concave, that is, curved inwards and straightened out to a greater extent towards the fovea. Note that the morphological operations described herein are textbook image-processing techniques. Curious readers are directed towards the highly accessible Hypermedia Image Processing Reference written by Fisher et al. at homepages.inf.ed.ac.uk/rbf/HIPR2/morops.htm. For the more technically minded, we kindly direct them towards the source code at github.com/fyii200/MyopiaRetinalFeatures, which contains reproducible details of the implementation and is freely/openly available.

CRAE (Knudtson),^37^ CRVE (Knudtson),^37^ vessel tortuosity^38^ and vessel FD (classic box-counting method)^39^ were computed using AutoMorph.^34^ Measurements based on AutoMorph segmentation were previously reported to have good to excellent agreement with those derived from segmentation done by expert annotators.^34^ Vessel FD was a global measure of vascular complexity and density (a larger value corresponded to increased complexity and density).^40^ We developed an automated image processing pipeline using the Python programming language to compute the concavity of the papillomacular arterial and venous arcades (left panel of Fig. 3). Briefly, the binary mask defining the artery or vein (whichever was applicable) was first cropped (horizontally) to around half of that of the original dimension, as the papillomacular arcade was observed to straighten out beyond that point. A series of morphological operations was then applied to remove the small vessels, before using circle Hough transform to extract the main vascular arcade.^41^ This was followed by another series of morphological operations to fill the small gaps in the foreground (vessel) pixels. A quadratic function (parabola) was then fitted to the skeleton (reduced to 1 pixel wide) of the vascular arcade using RANSAC, an iterative algorithm noted for its robustness to outliers.^42^ The absolute coefficient of the quadratic term represented vascular concavity, where a larger value indicated that the vascular arcade curved more inwards and appeared to straighten out as it coursed towards the fovea (right panel of Fig. 3). The median *r*-squared values were 0.92 (artery) and 0.93 (vein), suggesting that the fitted function described the overall course of the vascular arcade well (distribution of *r*-squared values is available as Supplementary S1).

Note that all dimensional metrics, including OD-fovea distance, CRAE, CRVE, OD major axis length and OD minor axis length, were expressed in pixels. Ocular magnification for these metrics was corrected using Littmann's formula^43^:

(2) $$\begin{array}{c} t=1.37\;\times \;q\times \;s\; \end{array}$$

where ***t*** denotes the true size of a parameter of interest, while ***s*** is the measured size. The variable ***q*** represents the ocular magnification factor, approximated using SER and CR (more details in Supplementary S2).

### Statistical Analyses

Multiple linear regression was first fitted using ordinary least squares (OLS) with all 12 retinal parameters simultaneously included as independent variables and SER as the dependent variable, controlling for age, sex and CR. Separate linear models were fitted for each eye and refractive group (−0.50 D as cutoff SER^44^). In OLS linear regression, retinal parameters were assumed to vary consistently and linearly with SER across the full phenotypic distribution of SER. In other words, the residuals were assumed to be normally distributed and have equal variance across the fitted (predicted) values. We visually inspected the normal quantile-quantile (Q-Q) and residuals versus fitted plots to assess if these assumptions were met. Multicollinearity (strong correlation between retinal parameters in the models) was checked with the variance inflation factor, treating 10 as the cutoff.^45^

In contrast to OLS linear regression, quantile regression (QR) is a flexible statistical modeling technique that allows for variability in the magnitude and direction of association between an independent variable and the dependent variable across the entire phenotypic distribution of the dependent variable.^46^ As such, it is not constrained by the OLS assumptions and can be especially valuable when the extremes of a phenotypic distribution (high ametropia) are of interest. QR was previously used to demonstrate that various genetic and environmental risk factors for myopia had larger effects on children with higher myopia.^47^ We also previously used QR to show that the rate of corneal biomechanical weakening increased as myopia increased.^48^ In this work, we applied QR to assess if the magnitude and direction of association between each retinal parameter and SER differed across 34 conditional quantiles of SER using the *Quantreg* package in R version 4.2.2 (R Core Team 2022, Vienna, Austria). The conditional quantiles ranged from 0.005 (most myopic) to 0.995 (most hyperopic). As before, all retinal parameters were simultaneously included as independent variables, with age, sex and CR as covariates.

Continuous independent variables and age were standardized (zero mean and unit standard deviation, SD) throughout to facilitate comparison and interpretation of the effect sizes of different retinal parameters. The significance level was set to 0.05. Note that in QR, correction of *P* values was not necessary because the “multiple” hypothesis testing for each retinal parameter (i.e., 34 tests, one per refractive quantile) was different from experiment-wise or family-wise multiple testing, as each unique eye belonged to only one refractive quantile (so only tested once in each model).^49^ Besides, it was the results of the *individual* tests (i.e., the magnitude of association in each quantile and whether this was statistically significant), rather than a single universal null hypothesis that all quantiles were not significant, that were of interest.^49^ The R, Python and MATLAB scripts used to perform the analyses (including image processing) described herein are freely and openly available at github.com/fyii200/MyopiaRetinalFeatures.

## Results

### Characteristics of Included Participants

Of the 23,064 eligible participants with a mean (SD; range) age of 53 (8; 40 to 69) years, 12,983 were female (56%) and 10,081 were male. Ninety-two percent of the participants were self-reportedly “British” (*n* = 19,440), “Irish” (*n* = 748), or with “Any other white background” (*n* = 1097). The mean SER and VA were −0.56 D (2.36; −16.88 to +9.22) and −0.11 logMAR (0.07; −0.42 to 0.00) for the right eye and −0.57 D (2.35; −22.99 to +8.58) and −0.11 logMAR (0.07; −0.48 to 0.00) for the left eye.

### Multiple Linear Regression

In myopes, all retinal parameters apart from OD-fovea angle were consistently (in both eyes) observed to be statistically significantly associated with SER (Table). Increasing myopia was associated with greater OD-fovea distance, larger OD, more tilted OD (towards the fovea), less circular OD, brighter fovea (more negative FPI), less tortuous vessels, less complex vasculature (lower FD), larger CRAE, larger CRVE and more concave papillomacular arterial/venous arcade. The effects of vessel FD, OD-fovea distance, CRVE, FPI, OD area and venous concavity appeared large, considering that one SD change in each parameter was independently (controlling for other parameters) associated with a 0.21 to 0.74 D change in SER.

**Table.** Multiple Linear Regression Fitted Using OLS, With 12 Retinal Parameters as Independent Variables (Simultaneously) and SER as the Dependent Variable

	Myopes (SER ≤−0.50 D)				NonMyopes (SER >−0.50 D)			
	RE (*n* = 6181)		LE (*n* = 5729)		RE (*n* = 11,539)		LE (*n* = 10,752)	
Parameter	Est (95% CI)	*P* Value	Est (95% CI)	*P* Value	Est (95% CI)	*P* Value	Est (95% CI)	*P* Value
Intercept	−2.99 (−3.06 to −2.93)	**<0.001**	−2.98 (−3.04 to −2.91)	**<0.001**	0.78 (0.76 to 0.81)	**<0.001**	0.76 (0.74 to 0.79)	**<0.001**
Male	0.03 (−0.07 to 0.12)	0.576	−0.03 (−0.13 to 0.08)	0.622	−0.10 (−0.14 to −0.06)	**<0.001**	−0.08 (−0.12 to −0.04)	**<0.001**
Age	0.16 (0.11 to 0.21)	**<0.001**	0.18 (0.13 to 0.23)	**<0.001**	0.36 (0.34 to 0.38)	**<0.001**	0.33 (0.31 to 0.35)	**<0.001**
CR	0.44 (0.39 to 0.49)	**<0.001**	0.41 (0.36 to 0.46)	**<0.001**	0.14 (0.12 to 0.16)	**<0.001**	0.10 (0.08 to 0.12)	**<0.001**
OD-fovea distance	−0.46 (−0.50 to −0.41)	**<0.001**	−0.42 (−0.47 to −0.37)	**<0.001**	−0.09 (−0.11 to −0.07)	**<0.001**	−0.03 (−0.06 to −0.01)	**0.003**
OD-fovea angle	0.07 (0.02 to 0.12)	**0.003**	−0.03 (−0.08 to 0.02)	0.185	0.01 (−0.01 to 0.03)	0.139	0.01 (−0.01 to 0.03)	0.388
OD orientation	0.16 (0.12 to 0.21)	**<0.001**	−0.15 (−0.20 to −0.11)	**<0.001**	−0.001 (−0.02 to 0.02)	0.929	−0.01 (−0.03 to 0.01)	0.456
FPI	−0.32 (−0.37 to −0.27)	**<0.001**	−0.23 (−0.29 to −0.18)	**<0.001**	−0.09 (−0.11 to −0.07)	**<0.001**	−0.11 (−0.14 to −0.09)	**<0.001**
OD ovality	−0.06 (−0.10 to −0.01)	**0.014**	−0.06 (−0.11 to −0.01)	**0.011**	−0.01 (−0.03 to 0.01)	0.213	−0.01 (−0.03 to 0.01)	0.206
OD area	−0.40 (−0.44 to −0.35)	**<0.001**	−0.38 (−0.44 to −0.33)	**<0.001**	−0.15 (−0.17 to −0.13)	**<0.001**	−0.13 (−0.15 to −0.11)	**<0.001**
CRAE	−0.11 (−0.16 to −0.05)	**<0.001**	−0.13 (−0.19 to −0.08)	**<0.001**	−0.06 (−0.09 to −0.04)	**<0.001**	−0.04 (−0.06 to −0.02)	**<0.001**
CRVE	−0.42 (−0.48 to −0.37)	**<0.001**	−0.46 (−0.52 to −0.41)	**<0.001**	−0.04 (−0.06 to −0.02)	**0.001**	−0.05 (−0.07 to −0.03)	**<0.001**
Vessel tortuosity	0.12 (0.08 to 0.17)	**<0.001**	0.12 (0.07 to 0.17)	**<0.001**	0.06 (0.05 to 0.08)	**<0.001**	0.03 (0.01 to 0.05)	**0.001**
Vessel FD	0.71 (0.65 to 0.76)	**<0.001**	0.74 (0.68 to 0.80)	**<0.001**	0.01 (−0.02 to 0.03)	0.588	−0.001 (−0.02 to 0.02)	0.898
Arterial concavity	−0.17 (−0.22 to −0.13)	**<0.001**	−0.24 (−0.29 to −0.18)	**<0.001**	−0.04 (−0.06 to −0.02)	**<0.001**	−0.02 (−0.04 to 0.00)	0.056
Venous concavity	−0.22 (−0.27 to −0.18)	**<0.001**	−0.21 (−0.26 to −0.16)	**<0.001**	−0.05 (−0.07 to −0.03)	**<0.001**	−0.05 (−0.07 to −0.03)	**<0.001**

Note that a more negative (or positive) OD orientation value suggests that the superior pole of the disc is tilted more towards the fovea in the right (or left) eye. Est, standardized parameter estimate (for all nonintercept terms, this refers to the standardized β-coefficient); LE, left eye; RE, right eye.

Bold values indicate that *P* < 0.05.

In nonmyopes, OD orientation, OD ovality, vessel FD, and arterial concavity were no longer consistently associated with SER. There was (still) insufficient evidence of a significant association between OD-fovea angle and SER in either eye. The direction of association for parameters that were significantly associated with SER was the same as before (in myopes). Compared to myopes, however, all retinal parameters had smaller associations with SER. There was no evidence of multicollinearity in both models (variance inflation factor ≤1.5 for all independent variables and covariates), but the normal Q-Q and residuals versus fitted plots indicated that the OLS assumptions were violated (Supplementary S3), suggesting that the associations could not be well described using conventional linear models.

### Quantile Regression

**Figure 4.**
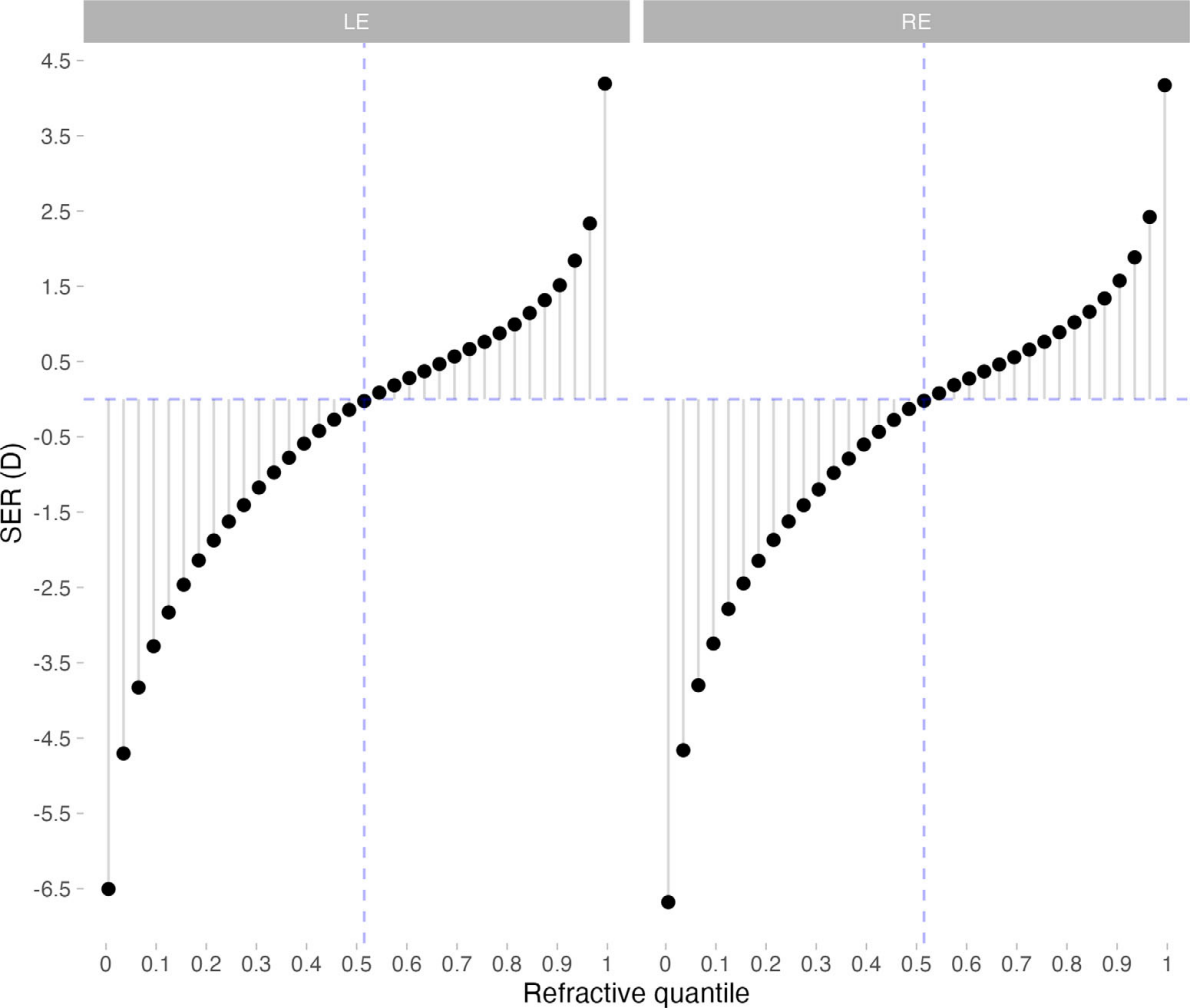
Intercept of quantile regression by refractive quantile (i.e., spherical equivalent refraction when all retinal parameters were set to their respective quantile-specific mean values), ranging from 0.005 (most myopic quantile) to 0.995 (most hyperopic quantile).

Figure 4 shows the intercept of QR regression by refractive quantile, that is, the SER when all retinal parameters and covariates were set to their respective quantile-specific mean values.

**Figure 5.**
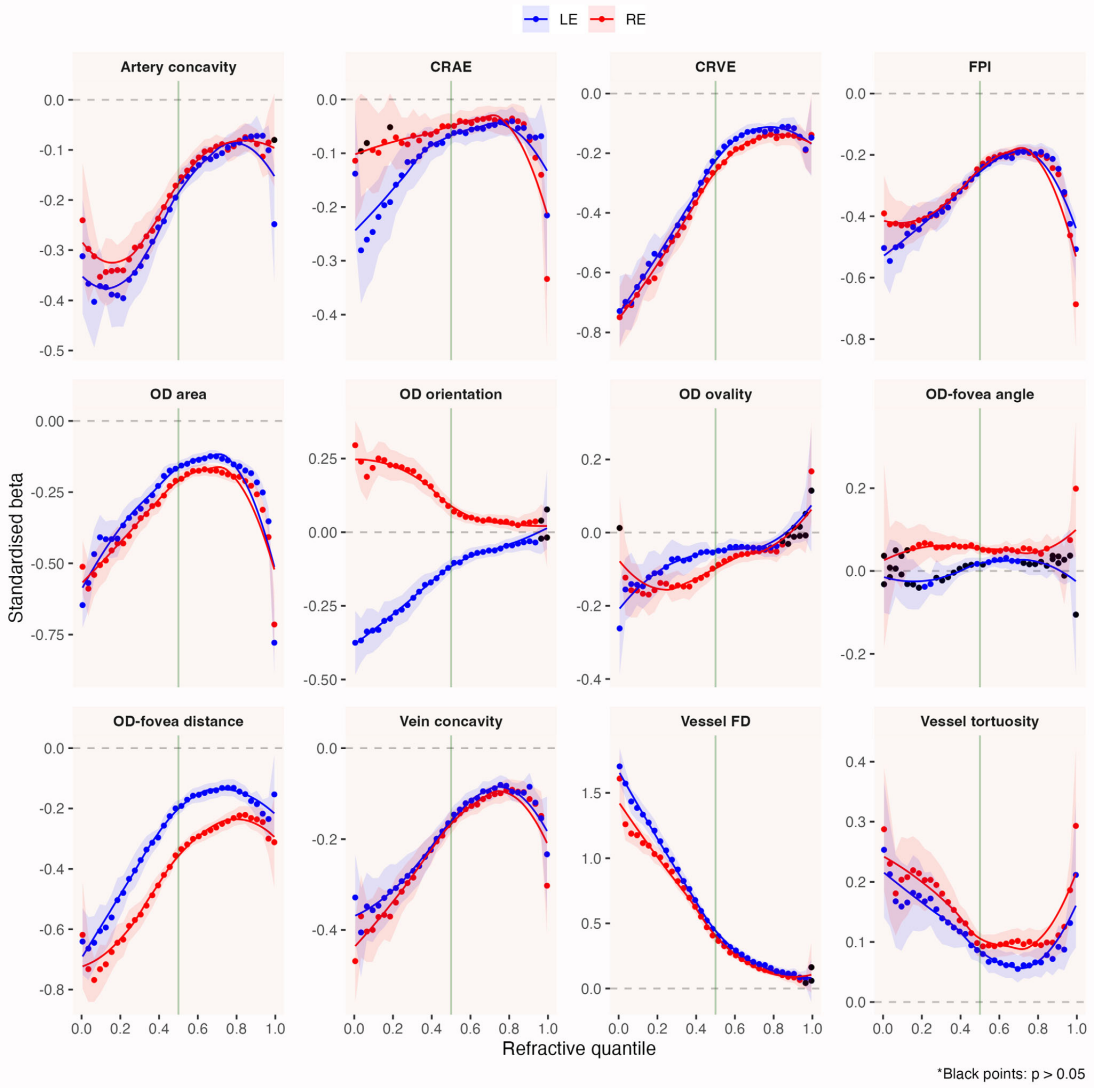
Standardized β-coefficient versus refractive quantile plots show how the magnitude of association between each retinal parameter and SER changes across refractive quantiles (right to left: high hyperopia to high myopia). A horizontal trendline across the full range of refractive quantiles (within a subplot) would suggest a linear relationship between that retinal parameter and SER, while a nonhorizontal trendline would indicate that its magnitude of association with SER changes depending on the severity of refractive error (nonlinear relationship). The *green vertical line* denotes refractive quantile corresponding to emmetropia, while the *dotted horizontal line* represents the line of null effect. *Colored points* represent statistically significant associations (*P* < 0.05). *Shaded region* represents the 95% CI. Regression results for each refractive quantile and eye in tabular format are available as Supplementary S4 and S5.

**Figure 6.**
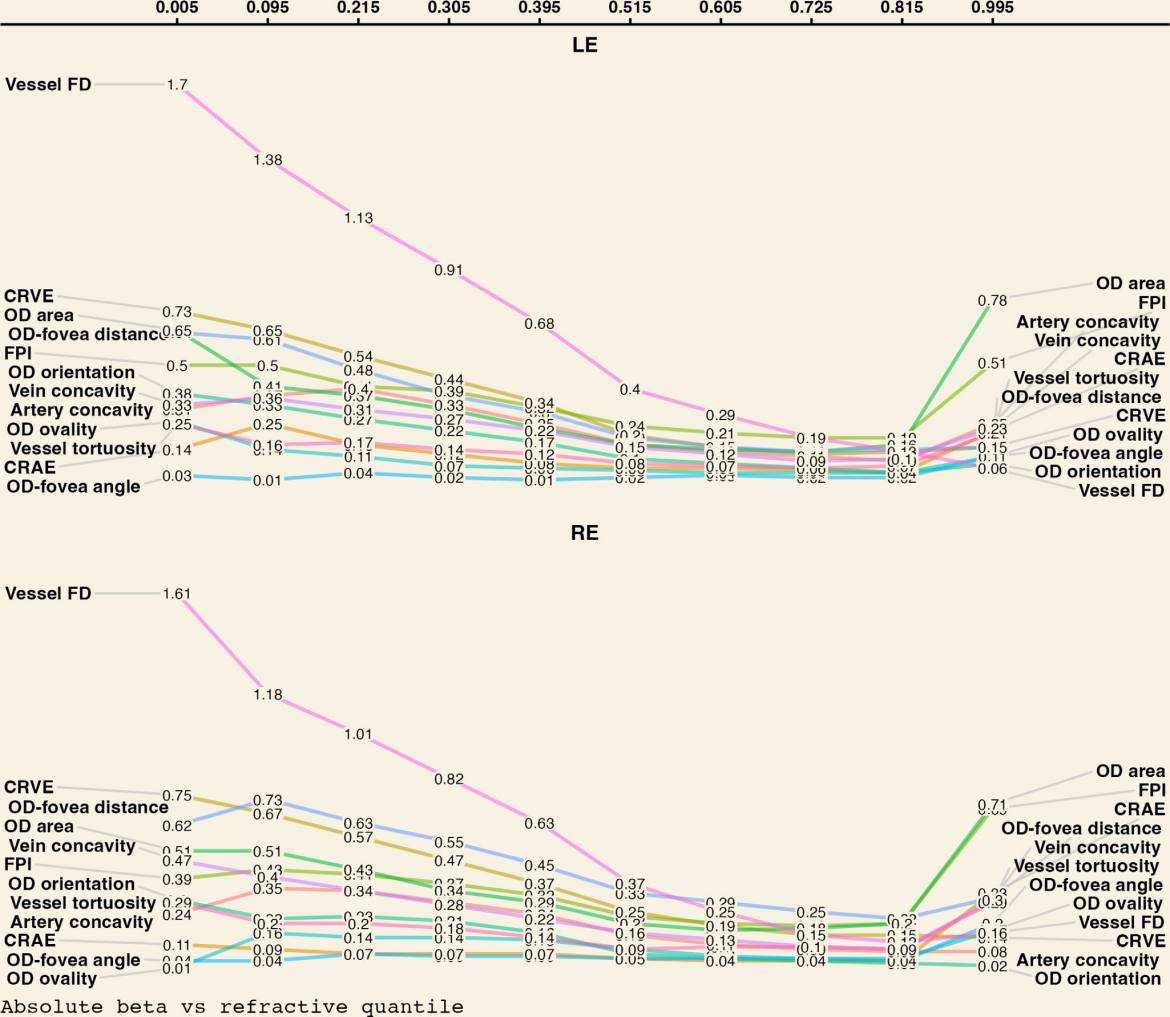
Absolute standardized magnitude of association (rounded values shown within the trendline) between each retinal parameter and spherical equivalent refraction from the most hyperopic quantile (furthest to the right) to the most myopic quantile (furthest to the left).

As can be seen in Figure 5, the magnitude of the standardized β-coefficient for almost all retinal parameters (except OD-fovea angle) *varied* systematically across the full range of refractive quantiles (regression results in tabular format are available as Supplementary S4 and S5). Each parameter is ranked based on its absolute magnitude of association with SER (by refractive quantile) in Figure 6. There was evidence that more negative SER was *nonlinearly* associated with greater OD-fovea distance, larger OD, less circular OD, less vertically orientated OD (tilted towards the fovea), brighter fovea, less tortuous vessels, less complex vasculature, larger CRAE, larger CRVE, and more concave papillomacular arterial/venous arcade (Fig. 5). Note that the increase in CRVE appeared to be more pronounced than CRAE, and thus the arteriovenous ratio was observed to decrease as SER decreased (Supplementary S6).

Three types of trends can generally be observed as SER progressed from the most hyperopic quantile to the most myopic quantile (from right to left in Figs. 5 and 6). The most common trend showed a relatively small or no association in hyperopia to a progressively larger association with more negative SER, which included such parameters as papillomacular arterial/venous concavity, CRVE, OD orientation, OD ovality, OD-fovea distance and vessel FD. To illustrate, vessel FD progressed from having no association with SER in the most hyperopic quantile to having the largest association relative to other parameters in low myopia (quantile corresponding to −0.60 D), in both right (standardized β, 0.63; 95% confidence interval [CI], 0.58–0.68; *P* < 0.001) and left (standardized β, 0.68; 95% CI, 0.63–0.73; *P* < 0.001) eyes. Its effect size continued to increase with increasing myopia, so much so that in the most myopic quantile, each SD reduction in FD was observed to be associated with 1.61 D (95% CI, 1.40–1.82; *P* < 0.001) and 1.70 D (95% CI, 1.56–1.84; *P* < 0.001) higher myopia. Likewise, OD orientation was not associated with SER near the hyperopic end of the refractive error distribution, but in the most myopic quantile, every SD increase in tilt angle (superior pole pointing towards the fovea) was associated with 0.30 D (right eye; 95% CI, 0.21–0.38; *P* < 0.001) and 0.38 D (left eye; 95% CI, 0.27–0.48; *P* < 0.001) higher myopia.

The second most common trend was characterized by a U (or inverted-U) shape, in which the magnitude of association changed from being large in hyperopia to small in emmetropia, before increasing again in myopia (i.e., FPI, OD area and vessel tortuosity). For example, while every SD increase in OD area was associated with a 0.71 D (right eye; 95% CI, 0.60–0.83; *P* < 0.001) and 0.51 D (right eye; 95% CI, 0.42–0.60; *P* < 0.001) decrease in SER in the most hyperopic and myopic quantiles, respectively, a similar increase in OD area was only associated with a 0.20 D (95% CI, 0.18–0.22; *P* < 0.001) and 0.16 D (95% CI, 0.18–0.14; *P* < 0.001) decrease in SER near emmetropia.

In contrast to the two nonlinear trends above, the third trend was characterized by a constant (practically linear) association of negligible magnitude, with OD-fovea angle being the only example among all parameters considered herein. While there was some statistical evidence of a positive association between OD-fovea angle and SER away from the extreme ends of the refractive error distribution, every SD decrease in OD-fovea angle (increased vertical distance between OD and fovea) was associated with less than a 0.10 D decrease in SER in almost all refractive quantiles, which was disproportionately smaller than the variation in (horizontal) OD-fovea distance, considering that SER decreased by as much as 0.70 D for every SD increase in OD-fovea distance in high myopia (Fig. 5).

## Discussion

Several possibly interrelated topographical, morphological, and geometrical changes could be observed at the posterior pole as SER progressed in the myopic direction. The relative position of OD and fovea appeared altered predominantly in the horizontal direction, with a significant increase in OD-fovea distance as myopia increased but not OD-fovea angle (practically unimportant variation). This observation of anisotropic (largely horizontal) stretching coincided with the retinal vessels appearing stretched in the temporal direction as they coursed towards the fovea, as indicated by a more concave papillomacular arterial/venous arcade. The retinal vasculature also became less tortuous and less complex/dense overall. At the same time, the OD became larger, less circular and increasingly tilted with its superior pole pointing towards the fovea. The fovea, interestingly, appeared brighter with increasing myopia, while CRAE and CRVE were observed to increase. Videos showing these changes are available at github.com/fyii200/MyopiaRetinalFeatures or as Supplementary Videos S1 to S4.

Importantly, we found evidence that these retinal changes, with the sole exception of OD-fovea angle, occurred in a highly nonlinear fashion across refractive error. In myopia, these changes were found to increase exponentially (in terms of magnitude) with more negative SER. If one assumes that the magnitude of these changes reflects the degree of retinal stretching at the posterior pole, then our findings may explain why the risk of myopic complications such as myopic maculopathy increases exponentially (nonlinearly) with increasing myopia.^22^ A possible underlying mechanism for these nonlinear changes is the nonuniform or nonconstant variation in ocular expansion patterns across refractive error. Previous studies investigating eye shape in children or adults using three-dimensional magnetic resonance imaging techniques found that the posterior segment of the eye became more prolate (decreased oblateness) in myopia, with the posterior pole becoming increasingly steeper centrally than peripherally, giving it a more “pointed” appearance.^50^ It may be that this increase in ocular prolateness, that is, increasing deviation from a uniform/global expansion of the posterior segment, induces progressively more pronounced changes per diopter decrease in SER at the posterior pole, which are reflected contemporaneously in fundus images. If so, characterising fundus changes in myopic eyes may facilitate personalized risk prediction for myopic complications affecting the posterior pole, especially myopic maculopathy and myopic traction maculopathy because posterior eye shape has been implicated in the frequency or prognosis of these diseases.^51^^,^^52^

While the overall (global) pattern of ocular expansion is relatively well studied in myopia,^50^ the nature of myopic stretching at the posterior pole is rarely explored, that is, whether it is isotropic (uniform) or anisotropic (favoring one orientation). An early psychophysical study found that under extrafoveal viewing conditions, the separation of two stimuli orientated *vertically* was misperceived to be larger than the same stimuli orientated *horizontally*, and the degree of this orientational misperception was evidently larger in myopes compared to emmetropes.^53^ It was suggested that during myopic axial growth, the posterior pole (and, by extension, the neural unit arrays) would stretch preferentially in the horizontal orientation, causing *vertical* stimuli to span even more neural units than horizontal stimuli, which in turn gave rise to increasingly large overestimation of vertical separation in myopes.^53^ This, along with our more direct finding of anisotropic (horizontal temporal) posterior pole stretching in myopia, is consistent with clinical observations that peripapillary atrophy tends to extend temporally. In addition, lacquer cracks in pathologic myopia are also found most frequently in the temporal retinal quadrant.^54^

In what follows, we discuss the changes in each retinal parameter in greater detail. Consistent with previous studies involving Chinese participants, we found evidence that more negative SER was associated with greater OD-fovea distance,^23^^,^^55^ while there was relatively little variation in OD-fovea angle.^24^^,^^55^ Jonas et al.^23^ reported that the distance between Bruch's membrane opening (BMO) and fovea—that is, OD-fovea distance without the width of peripapillary β/γ zone—increased with greater AL in shorter eyes (AL <23.5 mm) but not in longer eyes. In longer eyes, however, they found that OD-fovea distance increased with larger β/γ zone, leading to the conclusion that OD-fovea elongation in myopia was largely driven by β/γ zone expansion. Using optical coherence tomography (OCT), Jonas et al.^56^ also found a stronger positive association between γ zone width and AL (*r* = 0.76, *P* < 0.001) relative to that between BMO-fovea length and AL (*r* = 0.13, *P* = 0.02) in a Chinese sample that was myopic on average (around −3.00 D). In light of this, our finding that OD-fovea distance was more strongly associated with SER in higher myopia may be explained by the expansion of the β/γ zone as the posterior segment became increasingly prolate, above and beyond a more organic or physiologic increase in BMO-fovea distance seen in nonmyopic eyes.

In keeping with our findings, previous studies also reported more obliquely orientated OD in higher myopia, with the superior pole of the disc generally tilting towards the fovea (accounting for 67.0% to 85.5% of all obliquely orientated discs).^7^^,^^9^^,^^57^ It has been suggested that this is due to a stronger backward pull at the inferotemporal OD border during axial elongation, which may also give rise to a more oval (less circular) OD appearance.^3^ Our observation that the magnitude of association between OD area and SER varied in an inverted “U” shape may be explained as follows. With decreasing hyperopia (less positive SER), the rate of OD enlargement decreases as a result of increased obscuration of OD caused by a growing proportion of Bruch's membrane overhanging into the nasal intrapapillary compartment,^58^ changes that may be explicable by the anisotropic (horizontal temporal) stretching of the posterior pole described earlier. In myopia, as this posterior pole stretching becomes ever more pronounced, BMO enlarges and gradually uncovers more OD tissue and lamina cribrosa, causing the rate of OD enlargement to pick up again.^58^

To our knowledge, no work has directly examined the association between foveal brightness in fundus images and SER, although there has been an attempt to investigate changes in fundus reflectivity during myopia development in chickens.^59^ Our finding of a significant association was unlikely to have been confounded by the presence of macular pathology (e.g., myopic maculopathy, foveal hypoplasia) or nuclear sclerosis, given that we excluded eyes with ocular pathology or poor VA. Macular pigment optical density has previously been reported to be lower in eyes with greater AL or more negative SER.^60^^,^^61^ It may, therefore, be tempting to ascribe our finding to reduced macular pigment in myopic eyes, but this is far from certain because other studies failed to find a similar association,^62^^,^^63^ not to mention that fundus photography may not have the resolution required for changes in macular pigment to be detected (although this could be possible^64^ with the help of interference filters). Likewise, differences in foveal cone density^65^ also appear unlikely to be the reason because foveal cone-to-cone spacing is only around 3 microns on average compared with a resolution of ≥5 microns in conventional fundus imagng.^66^ A more plausible explanation is the variation in foveal morphology itself. The foveal pit becomes more curved with increasing myopia, as recently confirmed by a study analysing over 10,000 OCT scans in the UK Biobank.^67^ These changes, we hypothesize, could potentially have an effect on the frequency and/or brightness of the foveal reflex. In addition to this explanation, we cannot rule out the possibility that some of this association was due to selection bias inadvertently introduced by the exclusion of poor-quality images (e.g., perhaps centrally underexposed images with more negative SER were more likely to be rejected than similarly underexposed images with less negative SER, considering that the DL model that we used for image quality assessment might be more likely to reject “pathologic”-looking fundus photographs).^68^

The association between vessel tortuosity and refractive error has only been quantitatively examined by another study on diabetic participants from the Singapore Malay Eye Study (*N* = 2882).^17^ Consistent with their finding, more negative SER was associated with less tortuous vessels in healthy UK Biobank participants. Previous studies found that the angle between the supratemporal and inferotemporal arterial arcades decreased with increasing AL in Chinese high myopes.^25^^,^^26^^,^^69^ Our study suggests that such a trend is not unique to high myopes (and artery), as we found evidence of a significant (exponential) relationship between papillomacular arterial or venous concavity and SER, even in eyes with hyperopia.

In contrast to our findings, previous studies generally reported decreasing (not increasing) vascular calibre with increasing myopia.^12^^–^^15^ These studies, however, did not account for the effect of ocular magnification, so the reported association might have just been an optical artifact caused by the minification effect of myopia. Indeed, we found a *similar* direction of association to what these studies reported after repeating our analyses *without* correcting for the effect of magnification (Supplementary S7). Furthermore, both Cheung et al.^18^ and Wong et al.^19^ reported that the significant negative association between vessel calibre and myopia *disappeared* after they corrected for ocular magnification—although two other Singapore-based studies, one focusing on preschoolers^16^ and another on diabetic adults,^17^ still found a significant association after magnification correction. Considering that we are the only study that looked at healthy (e.g., no diabetes, hypertension) Caucasian adults with a significantly larger sample size (23,092 participants) than other studies (469 to 3654 participants), differences in general health, age, ethnicity, and/or sample size may be the reason for the discrepancy in results. It appears even from this short discussion that the association between retinal vessel calibre and refractive error is—in contrast to what might have been assumed—far from conclusive. Given that the overall retinal surface area increases with increasing myopia,^70^ coupled with a reduction in retinal vessel density, increasing CRAE and CRVE with decreasing SER may represent a compensatory response in *healthy* eyes to maintain normal retinal perfusion, although this remains a matter of conjecture until more work is carried out.

Our finding that vessel FD was positively associated with SER agrees with the Singapore Malay Eye Study^20^ and Blue Mountains Eye Study.^21^ The latter study^21^ found that the slope of this association became steeper around −4.00 D, although the authors emphasized that this needed to be corroborated by larger studies due to a very small number of high myopes included in their study (*N* = 29). In our much larger sample, we could ascertain that the relationship between vessel FD and SER was indeed not linear, with the rate of change increasing with increasing myopia, so much so that in high myopia, vessel FD was observed to have a disproportionately larger effect size than any other retinal parameter considered herein. Given that we only included healthy participants and controlled for age in our analyses, the significant association between lower FD and more negative SER was unlikely to have been confounded by vascular changes due to other systemic/ocular conditions^21^ or older age but the rarefaction of smaller retinal vessels in more myopic eyes (i.e., FD reflects how space-filling a structure is; a smaller value indicates less dense vasculature).^40^ However, it remains unclear if this represents a real biological effect or some kind of optical artifact, or a combination of both. The narrative surrounding the nature of this association appears to favor a biological effect based on the assumption that vessel FD is a dimensionless metric and not influenced by ocular magnification.^20^^,^^21^ While we agree that FD is indeed a dimensionless metric (not based on any specific scale), it may be erroneous to think that vessel FD in the context of refractive error is not influenced by ocular magnification. As much as a biological reduction in vascular density is possible, the minification effect seen in myopic eyes (which cannot be directly corrected for in the context of FD) may equally be likely to cause the smaller retinal vessels to be obscured. Further work is required to elucidate whether this association is (more) biological or optical in nature.

The present work is the largest study of its kind with more than 23,000 *healthy* participants analyzed. It is also unique in that we were able to consider various retinal parameters collectively and elucidate their independent associations (controlling for one another) with refractive error without the confounding effect of other ocular and systemic conditions. The use of a flexible regression technique also enabled us to gain greater insights into the nature of these associations across a wide range of refractive error, revealing that retinal alterations per diopter change in SER become more pronounced with increasing myopia. Although not a main focus of this work, the strong associations of a wide range of retinal parameters with refractive error also underscore the importance of SER adjustment in research interested in uncovering retinal phenotypes predictive of other ocular diseases while recognising that these associations are not linear. Finally, the wide range of retinal parameters derived from a huge number of eyes in this work shall enable the development of an end-to-end DL model capable of predicting these parameters directly from fundus images—thereby circumventing the need for complex, multistage pipelines reliant on accurate detection and segmentation of retinal features (such as vessels), which may not necessarily be robust to image quality issues. To this end, we will return all derived retinal parameters to the UK Biobank, making the data available to all existing and future researchers with access to the database.

One limitation of this study was the lack of AL information in our data set, so we were not able to completely exclude eyes with refractive ametropia. There is also some evidence that the relationship between SER and AL is not perfectly linear,^71^ although the degree of nonlinearity, we should stress, is disproportionately smaller than that of the associations between SER and the retinal parameters investigated herein. This limitation is also unlikely to have any significant impact on the overall findings, given that both myopia and hyperopia are predominantly axial in nature, not to mention that we excluded eyes with extreme CR and controlled for CR in all regression models. Another limitation is the potential influence of head tilt on OD-fovea angle, although the amount of compensatory ocular torsion due to head tilt is relatively small (±1 degree),^72^ and the presence/degree of head tilt is unlikely to be associated with refractive error. On a related note, eyes with nystagmus or strabismus were excluded, so our findings were also unlikely to be influenced by conditions affecting ocular motility. The magnification correction formula used in the present study (or any other existing formula for that matter) has intrinsic limitations because it assumes that the eye is rotationally symmetric with three surfaces (anterior cornea, anterior lens, and posterior lens) and that all ray angles are small (paraxial approximation), which are known to be false but useful for simplicity's sake.^73^ Recent work using a more anatomically correct nonsymmetric, four-surface model eye (additionally incorporating the posterior cornea)—coupled with real ray tracing—showed that although existing magnification correction methods were generally correct in eyes with emmetropia or lower levels of myopia, the error became noticeable (around 7.5%) in highly myopic eyes (−14.5 D).^73^ That said, in our study, dimensional metrics including CRVE, OD area and OD-fovea distance showed clear nonlinear changes with SER even in emmetropia and low ametropia (not explicable by the said limitations).

## Conclusions

Various retinal alterations indicative of an increasing (nonconstant) rate of posterior pole stretching are evident as SER increases in the myopic direction. This may explain why the risk of myopic complications increases exponentially, rather than linearly, with higher myopia. Changes in OD-fovea position, vascular topography and OD orientation collectively suggest that the posterior pole stretches predominantly (anisotropically) in the temporal horizontal direction. A thorough and quantitative characterisation of fundus changes may, we hypothesize, facilitate personalized risk prediction for sight-threatening myopic complications—significantly more so than refractive error or AL alone, considering that these one-dimensional measurements, that is, *point-to-point* biometrics pertaining to the distance between the cornea and fovea, do not directly mirror the extent of mechanical stretching *across* the posterior pole.

## Supplementary Material

Supplement 1

Supplement 2

Supplement 3

Supplement 4

Supplement 5

## Supplementary Material

**Supplementary Video S1**. Simulation of retinal changes (right eye) using the mean value of each retinal parameter (correcting for ocular magnification as appropriate) stratified by refractive error.

**Supplementary Video S2**. Simulation of retinal changes (left eye) using the mean value of each retinal parameter (correcting for ocular magnification as appropriate) stratified by refractive error.

**Supplementary Video S3**. Retinal changes across refractive error (right eye) created by averaging the segmentation masks.

**Supplementary Video S4**. Retinal changes across refractive error (left eye) created by averaging the segmentation masks.
