# Supplementary material for: Retinal Changes From Hyperopia to Myopia: Not All Diopters Are Created Equal
Source: Invest Ophthalmol Vis Sci. 2024 May 17;65(5):25. doi: 10.1167/iovs.65.5.25 (PMC11107950; doi:10.1167/iovs.65.5.25)
Supplement: Supplement 1 [file iovs-65-5-25_s001.pdf]

Supplementary Material

S1: Distribution of r-squared values of the fitted papillomacular arterial (left) and venous (right) parabolas.

Median r-squared value (artery): 0.92 (75% of the data ≥ 0.89)

Median r-squared value (vein): 0.93 (75% of the data ≥ 0.91)

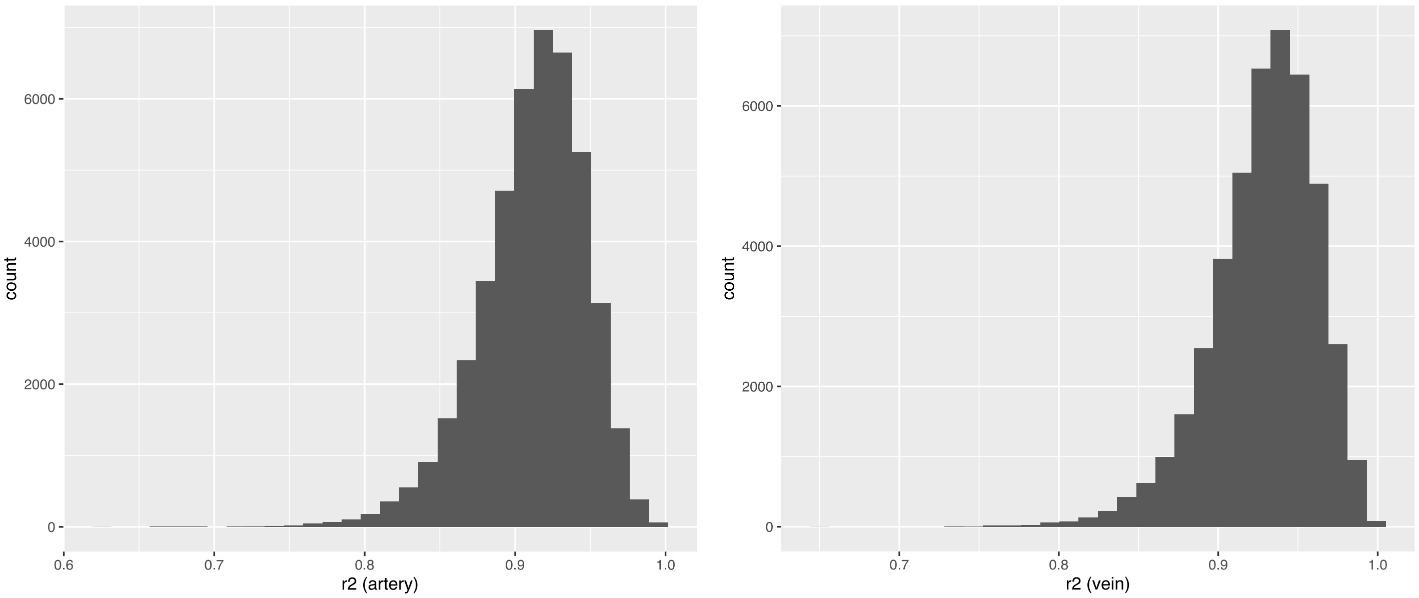

S2: Ocular magnification correction using Littmann's formula

The variable $\boldsymbol{q}$ in Littmann's formula relates to the optical dimensions of a given eye. It is approximated by the mean of corneal radius in the steepest and flattest meridians (CR), in mm, and spherical equivalent refraction (SER):

$$\boldsymbol{q} = \frac{\boldsymbol{a} \times \boldsymbol{SER}^2 - \boldsymbol{b} \times \boldsymbol{SER} + \boldsymbol{c}}{\mathbf{100}} \quad \textbf{(1)}$$

$$\boldsymbol{a} = \mathbf{0.01} + \mathbf{0.00236} \times (\boldsymbol{CR} - \mathbf{8}) \quad \textbf{(2)}$$

$$\boldsymbol{b} = \mathbf{0.6126} + \mathbf{0.0968} \times (\boldsymbol{CR} - \mathbf{8}) \quad \textbf{(3)}$$

$$\boldsymbol{c} = \mathbf{30.52} + \mathbf{2.57} \times (\boldsymbol{CR} - \mathbf{8}) \quad \textbf{(4)}$$

S3: Residuals vs Fitted plot and Normal Q-Q plot (multiple linear regression)

Normal Q−Q plot *ranks the observed residuals (difference between the actual and predicted SER) from smallest to largest on the y-axis while showing the theoretical residuals of the normal distribution on the x-axis. The plot should follow a straight (diagonal) line if the residuals are normally distributed (assumption 1).* ***Residuals vs Fitted plot*** *shows the observed residuals on the y-axis and the predicted SER on the x-axis. The residuals should be spread evenly along the y-axis if the variance of the residuals is equal across the range of predicted SER (assumption 2). Violation of these assumptions would suggest that the relationship between the derived retinal parameters and SER could not be modelled linearly across the range of refractive error.*

<u>Fitted to *myopic* eyes:</u>

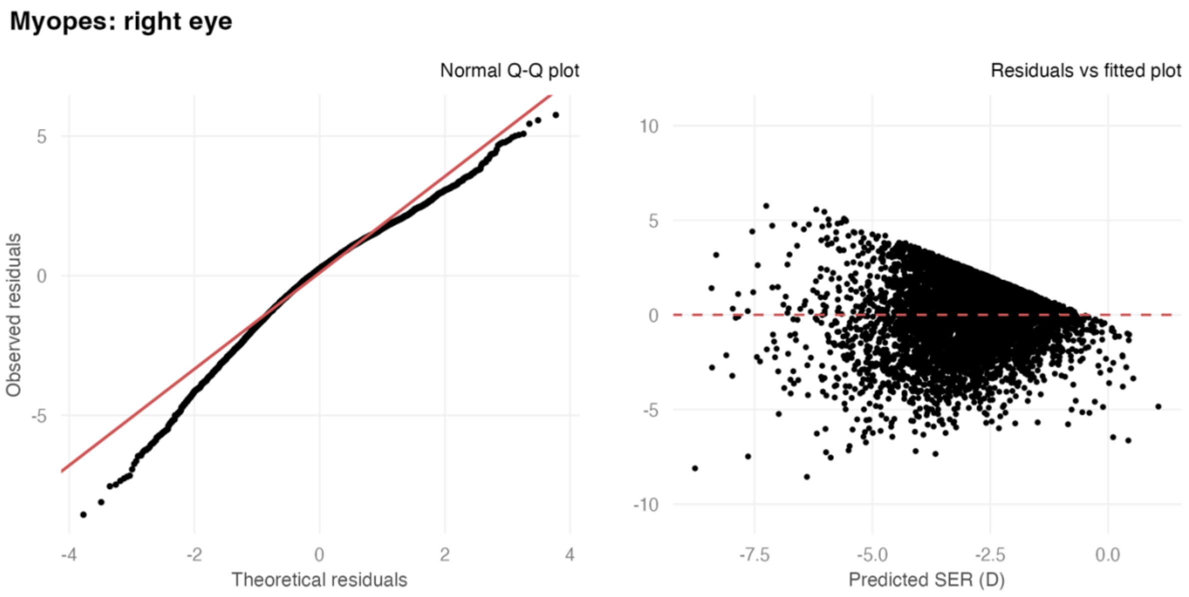

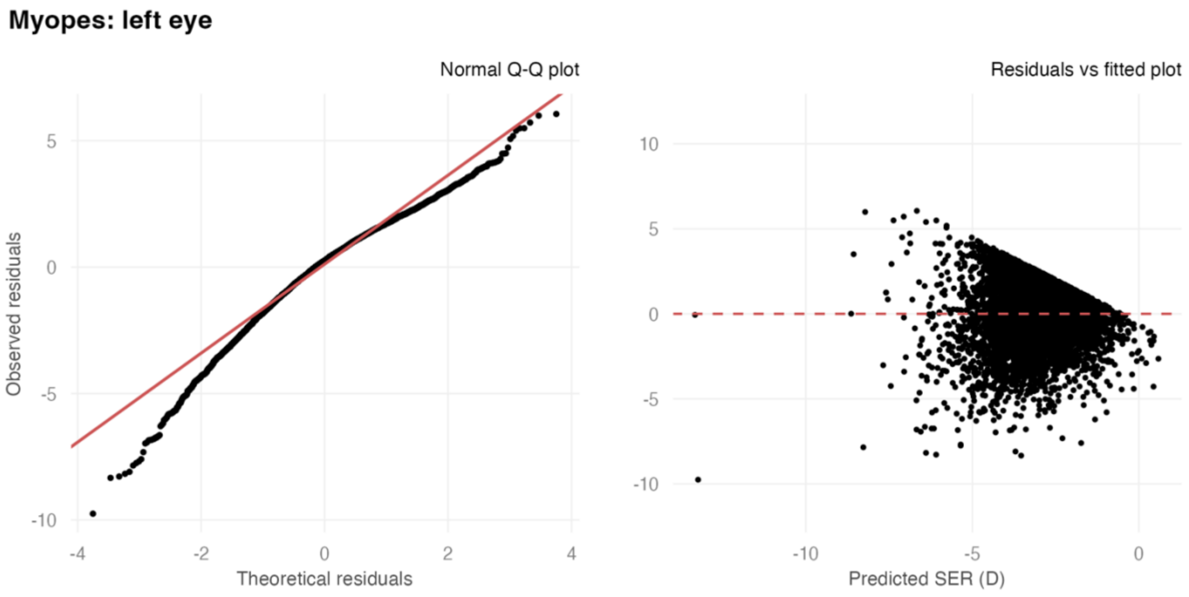

<u>Fitted to *non-myopic* eyes:</u>

Non-myopes: right eye

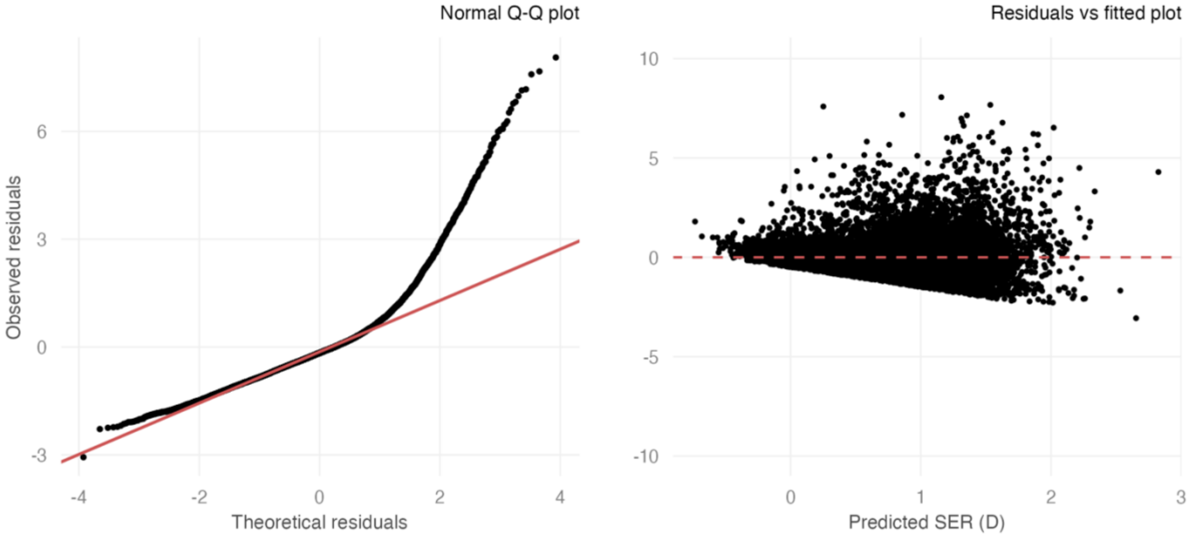

Non-myopes: left eye

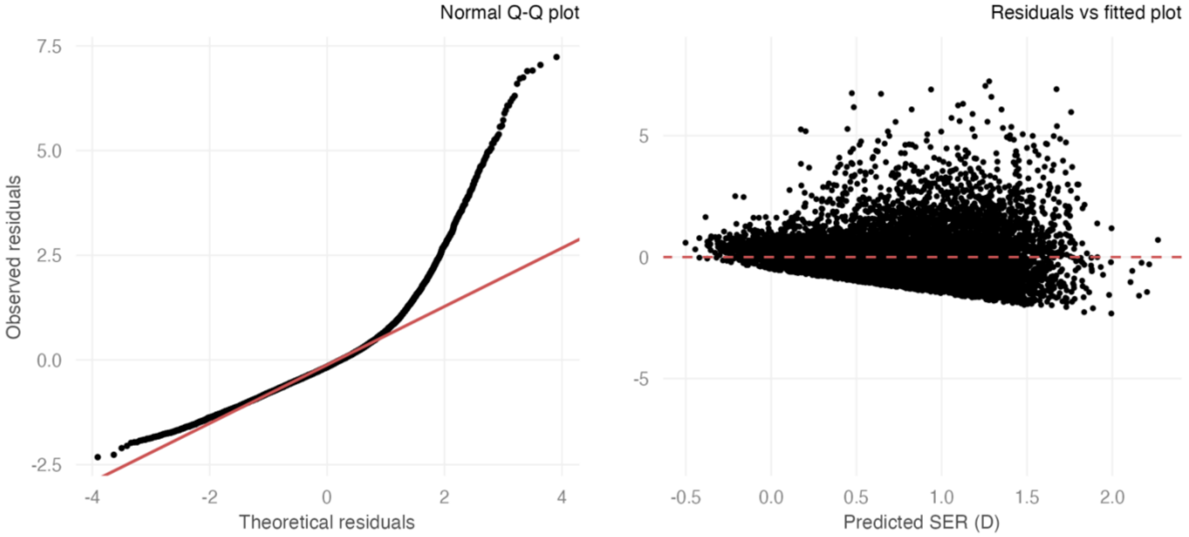

S4: Quantile regression results in long tabular format (right eye)

P-value is 0.000 when it is infinitesimally small. CR: corneal radius of curvature; OD: optic disc; FPI: foveal pixel intensity; CRAE: central retinal arteriolar equivalent; CRVE: central retinal venular equivalent; FD: fractal dimension.

Parameters	Quantile	Estimate	Standard error	P-value
**Intercept**	0.005	-6.680	0.122	0.000
	0.035	-4.662	0.066	0.000
	0.065	-3.799	0.054	0.000
	0.095	-3.245	0.050	0.000
	0.125	-2.786	0.044	0.000
	0.155	-2.446	0.040	0.000
	0.185	-2.146	0.036	0.000
	0.215	-1.869	0.037	0.000
	0.245	-1.623	0.033	0.000
	0.275	-1.407	0.031	0.000
	0.305	-1.198	0.032	0.000
	0.335	-0.978	0.031	0.000
	0.365	-0.789	0.028	0.000
	0.395	-0.602	0.027	0.000
	0.425	-0.433	0.025	0.000
	0.455	-0.274	0.024	0.000
	0.485	-0.129	0.020	0.000
	0.515	-0.019	0.017	0.246
	0.545	0.080	0.016	0.000
	0.575	0.189	0.015	0.000
	0.605	0.275	0.014	0.000
	0.635	0.370	0.015	0.000
	0.665	0.462	0.015	0.000
	0.695	0.558	0.015	0.000
	0.725	0.660	0.015	0.000
	0.755	0.763	0.016	0.000
	0.785	0.891	0.018	0.000
	0.815	1.023	0.018	0.000
	0.845	1.163	0.019	0.000
	0.875	1.340	0.021	0.000
	0.905	1.575	0.025	0.000
	0.935	1.885	0.032	0.000
	0.965	2.422	0.045	0.000
	0.995	4.172	0.118	0.000
**Male**	0.005	-0.054	0.084	0.518
	0.035	-0.017	0.094	0.855
	0.065	-0.141	0.075	0.058
	0.095	-0.200	0.071	0.005
	0.125	-0.212	0.065	0.001
	0.155	-0.219	0.056	0.000
	0.185	-0.212	0.055	0.000
	0.215	-0.217	0.053	0.000
	0.245	-0.199	0.046	0.000
	0.275	-0.186	0.043	0.000
	0.305	-0.171	0.042	0.000
	0.335	-0.197	0.038	0.000
	0.365	-0.180	0.032	0.000
	0.395	-0.195	0.027	0.000
	0.425	-0.197	0.022	0.000
	0.455	-0.175	0.020	0.000

	0.485	-0.171	0.020	0.000
	0.515	-0.163	0.018	0.000
	0.545	-0.151	0.016	0.000
	0.575	-0.161	0.017	0.000
	0.605	-0.150	0.019	0.000
	0.635	-0.151	0.020	0.000
	0.665	-0.146	0.020	0.000
	0.695	-0.142	0.020	0.000
	0.725	-0.141	0.021	0.000
	0.755	-0.135	0.023	0.000
	0.785	-0.152	0.023	0.000
	0.815	-0.162	0.024	0.000
	0.845	-0.150	0.027	0.000
	0.875	-0.129	0.030	0.000
	0.905	-0.138	0.032	0.000
	0.935	-0.104	0.043	0.016
	0.965	-0.034	0.057	0.553
	0.995	-0.078	0.121	0.517
**Age**	0.005	0.432	0.072	0.000
	0.035	0.449	0.050	0.000
	0.065	0.425	0.039	0.000
	0.095	0.406	0.037	0.000
	0.125	0.361	0.034	0.000
	0.155	0.387	0.029	0.000
	0.185	0.376	0.028	0.000
	0.215	0.383	0.027	0.000
	0.245	0.372	0.024	0.000
	0.275	0.357	0.022	0.000
	0.305	0.333	0.022	0.000
	0.335	0.329	0.019	0.000
	0.365	0.316	0.016	0.000
	0.395	0.308	0.014	0.000
	0.425	0.296	0.011	0.000
	0.455	0.283	0.010	0.000
	0.485	0.282	0.011	0.000
	0.515	0.285	0.009	0.000
	0.545	0.282	0.009	0.000
	0.575	0.287	0.010	0.000
	0.605	0.302	0.010	0.000
	0.635	0.313	0.011	0.000
	0.665	0.331	0.012	0.000
	0.695	0.352	0.012	0.000
	0.725	0.376	0.012	0.000
	0.755	0.398	0.013	0.000
	0.785	0.428	0.013	0.000
	0.815	0.467	0.014	0.000
	0.845	0.501	0.016	0.000
	0.875	0.537	0.017	0.000
	0.905	0.583	0.018	0.000
	0.935	0.595	0.022	0.000
	0.965	0.610	0.031	0.000
	0.995	0.641	0.065	0.000
**CR**	0.005	0.964	0.050	0.000
	0.035	0.863	0.047	0.000
	0.065	0.822	0.039	0.000
	0.095	0.794	0.037	0.000
	0.125	0.767	0.033	0.000
	0.155	0.725	0.028	0.000
	0.185	0.697	0.028	0.000
	0.215	0.694	0.027	0.000
	0.245	0.658	0.023	0.000
	0.275	0.622	0.021	0.000
	0.305	0.585	0.021	0.000

	0.335	0.554	0.019	0.000
	0.365	0.510	0.017	0.000
	0.395	0.479	0.014	0.000
	0.425	0.436	0.011	0.000
	0.455	0.390	0.010	0.000
	0.485	0.350	0.011	0.000
	0.515	0.330	0.008	0.000
	0.545	0.305	0.009	0.000
	0.575	0.294	0.009	0.000
	0.605	0.283	0.009	0.000
	0.635	0.266	0.010	0.000
	0.665	0.265	0.010	0.000
	0.695	0.250	0.010	0.000
	0.725	0.250	0.011	0.000
	0.755	0.250	0.012	0.000
	0.785	0.245	0.012	0.000
	0.815	0.250	0.012	0.000
	0.845	0.252	0.014	0.000
	0.875	0.258	0.015	0.000
	0.905	0.292	0.016	0.000
	0.935	0.335	0.022	0.000
	0.965	0.413	0.030	0.000
	0.995	0.622	0.071	0.000
**OD-fovea distance**	0.005	-0.619	0.091	0.000
	0.035	-0.732	0.056	0.000
	0.065	-0.768	0.037	0.000
	0.095	-0.733	0.042	0.000
	0.125	-0.716	0.032	0.000
	0.155	-0.676	0.033	0.000
	0.185	-0.645	0.031	0.000
	0.215	-0.634	0.029	0.000
	0.245	-0.589	0.026	0.000
	0.275	-0.570	0.025	0.000
	0.305	-0.551	0.022	0.000
	0.335	-0.522	0.021	0.000
	0.365	-0.487	0.021	0.000
	0.395	-0.455	0.018	0.000
	0.425	-0.420	0.012	0.000
	0.455	-0.393	0.015	0.000
	0.485	-0.355	0.011	0.000
	0.515	-0.334	0.010	0.000
	0.545	-0.319	0.010	0.000
	0.575	-0.301	0.011	0.000
	0.605	-0.292	0.011	0.000
	0.635	-0.281	0.013	0.000
	0.665	-0.272	0.012	0.000
	0.695	-0.263	0.012	0.000
	0.725	-0.251	0.013	0.000
	0.755	-0.242	0.015	0.000
	0.785	-0.232	0.014	0.000
	0.815	-0.223	0.016	0.000
	0.845	-0.222	0.015	0.000
	0.875	-0.232	0.019	0.000
	0.905	-0.236	0.021	0.000
	0.935	-0.245	0.023	0.000
	0.965	-0.300	0.028	0.000
	0.995	-0.312	0.077	0.000
**OD-fovea angle**	0.005	0.037	0.113	0.746
	0.035	0.008	0.049	0.870
	0.065	0.050	0.037	0.172
	0.095	0.036	0.040	0.368
	0.125	0.050	0.026	0.058
	0.155	0.055	0.026	0.034

	0.185	0.063	0.026	0.013
	0.215	0.067	0.022	0.002
	0.245	0.065	0.016	0.000
	0.275	0.057	0.019	0.002
	0.305	0.057	0.016	0.001
	0.335	0.058	0.013	0.000
	0.365	0.062	0.012	0.000
	0.395	0.059	0.009	0.000
	0.425	0.059	0.009	0.000
	0.455	0.062	0.005	0.000
	0.485	0.056	0.007	0.000
	0.515	0.051	0.006	0.000
	0.545	0.049	0.006	0.000
	0.575	0.046	0.009	0.000
	0.605	0.049	0.008	0.000
	0.635	0.053	0.011	0.000
	0.665	0.050	0.007	0.000
	0.695	0.047	0.009	0.000
	0.725	0.055	0.010	0.000
	0.755	0.053	0.014	0.000
	0.785	0.048	0.011	0.000
	0.815	0.041	0.015	0.007
	0.845	0.054	0.019	0.003
	0.875	0.037	0.020	0.060
	0.905	0.035	0.026	0.174
	0.935	-0.011	0.035	0.749
	0.965	0.075	0.025	0.002
	0.995	0.199	0.083	0.017
**OD orientation**	0.005	0.295	0.042	0.000
	0.035	0.240	0.051	0.000
	0.065	0.188	0.042	0.000
	0.095	0.218	0.030	0.000
	0.125	0.250	0.029	0.000
	0.155	0.245	0.031	0.000
	0.185	0.228	0.027	0.000
	0.215	0.225	0.026	0.000
	0.245	0.221	0.023	0.000
	0.275	0.212	0.022	0.000
	0.305	0.207	0.023	0.000
	0.335	0.188	0.023	0.000
	0.365	0.169	0.021	0.000
	0.395	0.155	0.019	0.000
	0.425	0.127	0.018	0.000
	0.455	0.109	0.015	0.000
	0.485	0.089	0.016	0.000
	0.515	0.070	0.013	0.000
	0.545	0.061	0.011	0.000
	0.575	0.052	0.011	0.000
	0.605	0.049	0.010	0.000
	0.635	0.043	0.011	0.000
	0.665	0.039	0.010	0.000
	0.695	0.043	0.010	0.000
	0.725	0.039	0.011	0.000
	0.755	0.035	0.011	0.002
	0.785	0.036	0.012	0.003
	0.815	0.030	0.013	0.018
	0.845	0.024	0.012	0.049
	0.875	0.030	0.015	0.046
	0.905	0.033	0.016	0.036
	0.935	0.036	0.018	0.050
	0.965	0.039	0.028	0.162
	0.995	-0.018	0.050	0.714
**FPI**	0.005	-0.391	0.063	0.000

	0.035	-0.426	0.052	0.000
	0.065	-0.424	0.039	0.000
	0.095	-0.430	0.041	0.000
	0.125	-0.429	0.032	0.000
	0.155	-0.427	0.030	0.000
	0.185	-0.413	0.029	0.000
	0.215	-0.407	0.028	0.000
	0.245	-0.400	0.024	0.000
	0.275	-0.397	0.024	0.000
	0.305	-0.371	0.022	0.000
	0.335	-0.352	0.020	0.000
	0.365	-0.331	0.019	0.000
	0.395	-0.319	0.014	0.000
	0.425	-0.304	0.014	0.000
	0.455	-0.280	0.011	0.000
	0.485	-0.247	0.008	0.000
	0.515	-0.228	0.010	0.000
	0.545	-0.214	0.010	0.000
	0.575	-0.207	0.011	0.000
	0.605	-0.201	0.010	0.000
	0.635	-0.198	0.011	0.000
	0.665	-0.196	0.011	0.000
	0.695	-0.188	0.011	0.000
	0.725	-0.189	0.011	0.000
	0.755	-0.194	0.013	0.000
	0.785	-0.201	0.012	0.000
	0.815	-0.199	0.014	0.000
	0.845	-0.210	0.016	0.000
	0.875	-0.243	0.017	0.000
	0.905	-0.263	0.020	0.000
	0.935	-0.329	0.025	0.000
	0.965	-0.463	0.029	0.000
	0.995	-0.686	0.072	0.000
**OD ovality**	0.005	0.013	0.044	0.772
	0.035	-0.123	0.052	0.018
	0.065	-0.157	0.038	0.000
	0.095	-0.158	0.037	0.000
	0.125	-0.167	0.020	0.000
	0.155	-0.169	0.030	0.000
	0.185	-0.157	0.030	0.000
	0.215	-0.137	0.025	0.000
	0.245	-0.140	0.012	0.000
	0.275	-0.149	0.021	0.000
	0.305	-0.144	0.021	0.000
	0.335	-0.147	0.016	0.000
	0.365	-0.147	0.019	0.000
	0.395	-0.130	0.018	0.000
	0.425	-0.124	0.014	0.000
	0.455	-0.115	0.013	0.000
	0.485	-0.098	0.013	0.000
	0.515	-0.088	0.011	0.000
	0.545	-0.078	0.011	0.000
	0.575	-0.071	0.012	0.000
	0.605	-0.063	0.011	0.000
	0.635	-0.059	0.011	0.000
	0.665	-0.058	0.012	0.000
	0.695	-0.053	0.012	0.000
	0.725	-0.051	0.011	0.000
	0.755	-0.048	0.013	0.000
	0.785	-0.049	0.012	0.000
	0.815	-0.052	0.015	0.001
	0.845	-0.033	0.015	0.030
	0.875	-0.031	0.017	0.063

	0.905	-0.011	0.019	0.549
	0.935	-0.009	0.017	0.592
	0.965	-0.008	0.025	0.757
	0.995	0.167	0.070	0.017
**OD area**	0.005	-0.512	0.047	0.000
	0.035	-0.589	0.049	0.000
	0.065	-0.540	0.039	0.000
	0.095	-0.506	0.038	0.000
	0.125	-0.490	0.036	0.000
	0.155	-0.455	0.031	0.000
	0.185	-0.430	0.031	0.000
	0.215	-0.430	0.029	0.000
	0.245	-0.404	0.026	0.000
	0.275	-0.370	0.025	0.000
	0.305	-0.341	0.024	0.000
	0.335	-0.327	0.022	0.000
	0.365	-0.299	0.018	0.000
	0.395	-0.292	0.017	0.000
	0.425	-0.262	0.015	0.000
	0.455	-0.229	0.014	0.000
	0.485	-0.210	0.012	0.000
	0.515	-0.203	0.011	0.000
	0.545	-0.187	0.010	0.000
	0.575	-0.176	0.010	0.000
	0.605	-0.175	0.010	0.000
	0.635	-0.170	0.011	0.000
	0.665	-0.174	0.011	0.000
	0.695	-0.175	0.011	0.000
	0.725	-0.182	0.011	0.000
	0.755	-0.188	0.011	0.000
	0.785	-0.196	0.011	0.000
	0.815	-0.196	0.012	0.000
	0.845	-0.211	0.011	0.000
	0.875	-0.228	0.013	0.000
	0.905	-0.258	0.014	0.000
	0.935	-0.312	0.016	0.000
	0.965	-0.408	0.023	0.000
	0.995	-0.714	0.057	0.000
**CRAE**	0.005	-0.114	0.057	0.045
	0.035	-0.096	0.055	0.079
	0.065	-0.081	0.042	0.054
	0.095	-0.094	0.041	0.023
	0.125	-0.099	0.037	0.008
	0.155	-0.079	0.033	0.017
	0.185	-0.052	0.032	0.106
	0.215	-0.071	0.031	0.021
	0.245	-0.081	0.024	0.001
	0.275	-0.083	0.025	0.001
	0.305	-0.067	0.025	0.008
	0.335	-0.076	0.023	0.001
	0.365	-0.064	0.020	0.001
	0.395	-0.065	0.014	0.000
	0.425	-0.060	0.014	0.000
	0.455	-0.050	0.011	0.000
	0.485	-0.050	0.012	0.000
	0.515	-0.049	0.011	0.000
	0.545	-0.040	0.011	0.000
	0.575	-0.042	0.011	0.000
	0.605	-0.044	0.011	0.000
	0.635	-0.038	0.012	0.002
	0.665	-0.036	0.012	0.002
	0.695	-0.035	0.012	0.003
	0.725	-0.038	0.012	0.002

	0.755	-0.039	0.013	0.003
	0.785	-0.041	0.013	0.002
	0.815	-0.035	0.014	0.012
	0.845	-0.040	0.016	0.010
	0.875	-0.046	0.016	0.005
	0.905	-0.076	0.017	0.000
	0.935	-0.108	0.022	0.000
	0.965	-0.140	0.030	0.000
	0.995	-0.334	0.065	0.000
**CRVE**	0.005	-0.749	0.052	0.000
	0.035	-0.712	0.056	0.000
	0.065	-0.709	0.043	0.000
	0.095	-0.674	0.043	0.000
	0.125	-0.617	0.038	0.000
	0.155	-0.631	0.034	0.000
	0.185	-0.619	0.033	0.000
	0.215	-0.570	0.031	0.000
	0.245	-0.525	0.027	0.000
	0.275	-0.495	0.026	0.000
	0.305	-0.475	0.026	0.000
	0.335	-0.448	0.024	0.000
	0.365	-0.415	0.019	0.000
	0.395	-0.367	0.018	0.000
	0.425	-0.326	0.015	0.000
	0.455	-0.291	0.013	0.000
	0.485	-0.266	0.014	0.000
	0.515	-0.245	0.011	0.000
	0.545	-0.230	0.011	0.000
	0.575	-0.201	0.009	0.000
	0.605	-0.186	0.012	0.000
	0.635	-0.182	0.012	0.000
	0.665	-0.167	0.012	0.000
	0.695	-0.160	0.012	0.000
	0.725	-0.148	0.013	0.000
	0.755	-0.141	0.014	0.000
	0.785	-0.138	0.013	0.000
	0.815	-0.150	0.014	0.000
	0.845	-0.142	0.016	0.000
	0.875	-0.139	0.017	0.000
	0.905	-0.142	0.018	0.000
	0.935	-0.148	0.023	0.000
	0.965	-0.190	0.031	0.000
	0.995	-0.138	0.068	0.043
**Vessel tortuosity**	0.005	0.288	0.053	0.000
	0.035	0.230	0.048	0.000
	0.065	0.181	0.036	0.000
	0.095	0.204	0.036	0.000
	0.125	0.208	0.033	0.000
	0.155	0.219	0.029	0.000
	0.185	0.214	0.028	0.000
	0.215	0.203	0.027	0.000
	0.245	0.203	0.023	0.000
	0.275	0.195	0.021	0.000
	0.305	0.181	0.022	0.000
	0.335	0.166	0.020	0.000
	0.365	0.153	0.017	0.000
	0.395	0.136	0.014	0.000
	0.425	0.131	0.011	0.000
	0.455	0.115	0.010	0.000
	0.485	0.098	0.011	0.000
	0.515	0.093	0.010	0.000
	0.545	0.094	0.009	0.000
	0.575	0.093	0.010	0.000

	0.605	0.096	0.010	0.000
	0.635	0.097	0.011	0.000
	0.665	0.100	0.011	0.000
	0.695	0.102	0.011	0.000
	0.725	0.096	0.011	0.000
	0.755	0.100	0.012	0.000
	0.785	0.096	0.012	0.000
	0.815	0.095	0.013	0.000
	0.845	0.099	0.013	0.000
	0.875	0.099	0.015	0.000
	0.905	0.111	0.016	0.000
	0.935	0.125	0.021	0.000
	0.965	0.186	0.027	0.000
	0.995	0.293	0.066	0.000
**Vessel FD**	0.005	1.610	0.105	0.000
	0.035	1.259	0.063	0.000
	0.065	1.188	0.039	0.000
	0.095	1.176	0.047	0.000
	0.125	1.116	0.040	0.000
	0.155	1.097	0.038	0.000
	0.185	1.032	0.036	0.000
	0.215	1.006	0.035	0.000
	0.245	0.944	0.031	0.000
	0.275	0.897	0.031	0.000
	0.305	0.824	0.032	0.000
	0.335	0.770	0.030	0.000
	0.365	0.696	0.027	0.000
	0.395	0.628	0.025	0.000
	0.425	0.551	0.022	0.000
	0.455	0.470	0.020	0.000
	0.485	0.407	0.019	0.000
	0.515	0.365	0.016	0.000
	0.545	0.325	0.015	0.000
	0.575	0.276	0.015	0.000
	0.605	0.254	0.015	0.000
	0.635	0.226	0.016	0.000
	0.665	0.201	0.016	0.000
	0.695	0.179	0.016	0.000
	0.725	0.154	0.016	0.000
	0.755	0.141	0.016	0.000
	0.785	0.132	0.016	0.000
	0.815	0.121	0.018	0.000
	0.845	0.102	0.019	0.000
	0.875	0.089	0.021	0.000
	0.905	0.084	0.022	0.000
	0.935	0.065	0.030	0.028
	0.965	0.042	0.022	0.060
	0.995	0.163	0.098	0.097
**Artery concavity**	0.005	-0.240	0.058	0.000
	0.035	-0.298	0.054	0.000
	0.065	-0.312	0.031	0.000
	0.095	-0.353	0.024	0.000
	0.125	-0.344	0.035	0.000
	0.155	-0.342	0.032	0.000
	0.185	-0.340	0.025	0.000
	0.215	-0.340	0.031	0.000
	0.245	-0.319	0.025	0.000
	0.275	-0.295	0.025	0.000
	0.305	-0.291	0.026	0.000
	0.335	-0.272	0.024	0.000
	0.365	-0.261	0.021	0.000
	0.395	-0.237	0.018	0.000
	0.425	-0.214	0.015	0.000

	0.455	-0.191	0.016	0.000
	0.485	-0.171	0.013	0.000
	0.515	-0.155	0.013	0.000
	0.545	-0.141	0.012	0.000
	0.575	-0.125	0.011	0.000
	0.605	-0.115	0.012	0.000
	0.635	-0.103	0.012	0.000
	0.665	-0.100	0.012	0.000
	0.695	-0.093	0.012	0.000
	0.725	-0.089	0.007	0.000
	0.755	-0.100	0.013	0.000
	0.785	-0.093	0.014	0.000
	0.815	-0.081	0.015	0.000
	0.845	-0.074	0.015	0.000
	0.875	-0.079	0.014	0.000
	0.905	-0.088	0.015	0.000
	0.935	-0.113	0.021	0.000
	0.965	-0.085	0.032	0.008
	0.995	-0.080	0.047	0.090
**Vein concavity**	0.005	-0.469	0.046	0.000
	0.035	-0.370	0.046	0.000
	0.065	-0.403	0.035	0.000
	0.095	-0.400	0.036	0.000
	0.125	-0.372	0.034	0.000
	0.155	-0.367	0.028	0.000
	0.185	-0.370	0.029	0.000
	0.215	-0.340	0.029	0.000
	0.245	-0.316	0.025	0.000
	0.275	-0.297	0.023	0.000
	0.305	-0.285	0.024	0.000
	0.335	-0.260	0.022	0.000
	0.365	-0.239	0.018	0.000
	0.395	-0.224	0.017	0.000
	0.425	-0.205	0.012	0.000
	0.455	-0.192	0.012	0.000
	0.485	-0.170	0.013	0.000
	0.515	-0.158	0.011	0.000
	0.545	-0.144	0.009	0.000
	0.575	-0.134	0.011	0.000
	0.605	-0.127	0.010	0.000
	0.635	-0.124	0.011	0.000
	0.665	-0.112	0.011	0.000
	0.695	-0.106	0.011	0.000
	0.725	-0.101	0.011	0.000
	0.755	-0.099	0.012	0.000
	0.785	-0.092	0.012	0.000
	0.815	-0.092	0.012	0.000
	0.845	-0.096	0.013	0.000
	0.875	-0.097	0.013	0.000
	0.905	-0.112	0.014	0.000
	0.935	-0.123	0.018	0.000
	0.965	-0.149	0.027	0.000
	0.995	-0.303	0.054	0.000

S5: Quantile regression results in long tabular format (left eye)

P-value is 0.000 when it is infinitesimally small. CR: corneal radius of curvature; OD: optic disc; FPI: foveal pixel intensity; CRAE: central retinal arteriolar equivalent; CRVE: central retinal venular equivalent; FD: fractal dimension.

Parameters	Quantile	Estimate	Standard error	P-value
**Intercept**	0.005	-6.505	0.121	0.000
	0.035	-4.705	0.060	0.000
	0.065	-3.829	0.057	0.000
	0.095	-3.280	0.053	0.000
	0.125	-2.831	0.045	0.000
	0.155	-2.463	0.044	0.000
	0.185	-2.140	0.040	0.000
	0.215	-1.875	0.036	0.000
	0.245	-1.624	0.034	0.000
	0.275	-1.405	0.034	0.000
	0.305	-1.173	0.034	0.000
	0.335	-0.972	0.031	0.000
	0.365	-0.777	0.031	0.000
	0.395	-0.589	0.027	0.000
	0.425	-0.421	0.025	0.000
	0.455	-0.270	0.023	0.000
	0.485	-0.139	0.020	0.000
	0.515	-0.023	0.019	0.221
	0.545	0.089	0.016	0.000
	0.575	0.186	0.016	0.000
	0.605	0.282	0.016	0.000
	0.635	0.372	0.015	0.000
	0.665	0.469	0.016	0.000
	0.695	0.568	0.016	0.000
	0.725	0.667	0.015	0.000
	0.755	0.762	0.016	0.000
	0.785	0.878	0.016	0.000
	0.815	0.995	0.019	0.000
	0.845	1.145	0.020	0.000
	0.875	1.317	0.021	0.000
	0.905	1.515	0.025	0.000
	0.935	1.841	0.031	0.000
	0.965	2.336	0.045	0.000
	0.995	4.193	0.130	0.000
**Male**	0.005	-0.426	0.127	0.001
	0.035	-0.040	0.092	0.667
	0.065	-0.130	0.080	0.103
	0.095	-0.144	0.075	0.054
	0.125	-0.231	0.061	0.000
	0.155	-0.249	0.063	0.000
	0.185	-0.252	0.059	0.000
	0.215	-0.233	0.050	0.000
	0.245	-0.206	0.046	0.000
	0.275	-0.203	0.044	0.000
	0.305	-0.194	0.042	0.000
	0.335	-0.188	0.037	0.000
	0.365	-0.180	0.034	0.000
	0.395	-0.174	0.027	0.000
	0.425	-0.161	0.021	0.000
	0.455	-0.151	0.021	0.000

	0.485	-0.138	0.017	0.000
	0.515	-0.130	0.018	0.000
	0.545	-0.134	0.018	0.000
	0.575	-0.133	0.020	0.000
	0.605	-0.143	0.020	0.000
	0.635	-0.131	0.020	0.000
	0.665	-0.140	0.021	0.000
	0.695	-0.140	0.021	0.000
	0.725	-0.128	0.021	0.000
	0.755	-0.120	0.021	0.000
	0.785	-0.118	0.021	0.000
	0.815	-0.110	0.024	0.000
	0.845	-0.121	0.026	0.000
	0.875	-0.119	0.027	0.000
	0.905	-0.088	0.036	0.014
	0.935	-0.098	0.033	0.003
	0.965	-0.045	0.059	0.447
	0.995	-0.267	0.129	0.039
**Age**	0.005	0.390	0.063	0.000
	0.035	0.437	0.047	0.000
	0.065	0.400	0.041	0.000
	0.095	0.385	0.039	0.000
	0.125	0.404	0.032	0.000
	0.155	0.392	0.033	0.000
	0.185	0.391	0.031	0.000
	0.215	0.360	0.025	0.000
	0.245	0.345	0.024	0.000
	0.275	0.335	0.022	0.000
	0.305	0.321	0.022	0.000
	0.335	0.302	0.019	0.000
	0.365	0.294	0.017	0.000
	0.395	0.287	0.014	0.000
	0.425	0.272	0.012	0.000
	0.455	0.269	0.010	0.000
	0.485	0.268	0.009	0.000
	0.515	0.266	0.009	0.000
	0.545	0.268	0.010	0.000
	0.575	0.271	0.011	0.000
	0.605	0.284	0.012	0.000
	0.635	0.300	0.012	0.000
	0.665	0.315	0.012	0.000
	0.695	0.335	0.013	0.000
	0.725	0.358	0.012	0.000
	0.755	0.388	0.013	0.000
	0.785	0.414	0.013	0.000
	0.815	0.435	0.015	0.000
	0.845	0.478	0.015	0.000
	0.875	0.514	0.016	0.000
	0.905	0.549	0.020	0.000
	0.935	0.574	0.018	0.000
	0.965	0.584	0.030	0.000
	0.995	0.515	0.070	0.000
**CR**	0.005	1.082	0.041	0.000
	0.035	0.814	0.046	0.000
	0.065	0.777	0.042	0.000
	0.095	0.725	0.038	0.000
	0.125	0.719	0.031	0.000
	0.155	0.676	0.032	0.000
	0.185	0.631	0.030	0.000
	0.215	0.621	0.025	0.000
	0.245	0.583	0.024	0.000
	0.275	0.563	0.021	0.000
	0.305	0.512	0.021	0.000

	0.335	0.475	0.018	0.000
	0.365	0.429	0.016	0.000
	0.395	0.396	0.013	0.000
	0.425	0.352	0.012	0.000
	0.455	0.321	0.011	0.000
	0.485	0.293	0.010	0.000
	0.515	0.265	0.008	0.000
	0.545	0.239	0.009	0.000
	0.575	0.229	0.010	0.000
	0.605	0.220	0.010	0.000
	0.635	0.210	0.010	0.000
	0.665	0.200	0.011	0.000
	0.695	0.194	0.011	0.000
	0.725	0.187	0.011	0.000
	0.755	0.186	0.011	0.000
	0.785	0.193	0.011	0.000
	0.815	0.190	0.013	0.000
	0.845	0.205	0.013	0.000
	0.875	0.211	0.013	0.000
	0.905	0.237	0.017	0.000
	0.935	0.273	0.017	0.000
	0.965	0.350	0.031	0.000
	0.995	0.666	0.072	0.000
**OD-fovea distance**	0.005	-0.641	0.056	0.000
	0.035	-0.664	0.053	0.000
	0.065	-0.645	0.045	0.000
	0.095	-0.606	0.045	0.000
	0.125	-0.594	0.037	0.000
	0.155	-0.561	0.039	0.000
	0.185	-0.504	0.037	0.000
	0.215	-0.479	0.030	0.000
	0.245	-0.433	0.025	0.000
	0.275	-0.405	0.029	0.000
	0.305	-0.371	0.027	0.000
	0.335	-0.336	0.023	0.000
	0.365	-0.313	0.018	0.000
	0.395	-0.296	0.014	0.000
	0.425	-0.257	0.016	0.000
	0.455	-0.226	0.014	0.000
	0.485	-0.200	0.012	0.000
	0.515	-0.192	0.011	0.000
	0.545	-0.171	0.011	0.000
	0.575	-0.158	0.012	0.000
	0.605	-0.155	0.013	0.000
	0.635	-0.149	0.014	0.000
	0.665	-0.142	0.014	0.000
	0.695	-0.140	0.013	0.000
	0.725	-0.132	0.013	0.000
	0.755	-0.131	0.014	0.000
	0.785	-0.132	0.015	0.000
	0.815	-0.144	0.015	0.000
	0.845	-0.153	0.014	0.000
	0.875	-0.175	0.013	0.000
	0.905	-0.186	0.023	0.000
	0.935	-0.217	0.024	0.000
	0.965	-0.235	0.037	0.000
	0.995	-0.153	0.067	0.022
**OD-fovea angle**	0.005	-0.032	0.035	0.364
	0.035	-0.015	0.046	0.753
	0.065	0.006	0.034	0.865
	0.095	-0.008	0.036	0.817
	0.125	-0.032	0.030	0.298
	0.155	-0.032	0.031	0.295

	0.185	-0.040	0.027	0.131
	0.215	-0.038	0.019	0.040
	0.245	-0.032	0.012	0.007
	0.275	-0.016	0.022	0.459
	0.305	-0.023	0.019	0.224
	0.335	-0.015	0.014	0.292
	0.365	-0.004	0.011	0.727
	0.395	0.005	0.009	0.554
	0.425	0.012	0.009	0.182
	0.455	0.015	0.009	0.088
	0.485	0.017	0.008	0.025
	0.515	0.016	0.008	0.061
	0.545	0.021	0.007	0.002
	0.575	0.026	0.008	0.001
	0.605	0.027	0.011	0.011
	0.635	0.032	0.013	0.014
	0.665	0.025	0.012	0.040
	0.695	0.024	0.011	0.034
	0.725	0.019	0.012	0.104
	0.755	0.016	0.013	0.227
	0.785	0.017	0.014	0.226
	0.815	0.022	0.016	0.168
	0.845	0.013	0.016	0.428
	0.875	0.029	0.017	0.099
	0.905	0.019	0.022	0.382
	0.935	0.027	0.031	0.375
	0.965	0.037	0.032	0.251
	0.995	-0.105	0.074	0.154
**OD orientation**	0.005	-0.375	0.055	0.000
	0.035	-0.368	0.038	0.000
	0.065	-0.337	0.040	0.000
	0.095	-0.334	0.038	0.000
	0.125	-0.332	0.030	0.000
	0.155	-0.301	0.034	0.000
	0.185	-0.293	0.030	0.000
	0.215	-0.273	0.025	0.000
	0.245	-0.263	0.025	0.000
	0.275	-0.248	0.027	0.000
	0.305	-0.223	0.024	0.000
	0.335	-0.202	0.023	0.000
	0.365	-0.179	0.019	0.000
	0.395	-0.171	0.017	0.000
	0.425	-0.156	0.014	0.000
	0.455	-0.137	0.014	0.000
	0.485	-0.121	0.012	0.000
	0.515	-0.104	0.012	0.000
	0.545	-0.103	0.011	0.000
	0.575	-0.085	0.013	0.000
	0.605	-0.077	0.012	0.000
	0.635	-0.070	0.011	0.000
	0.665	-0.067	0.011	0.000
	0.695	-0.062	0.011	0.000
	0.725	-0.062	0.011	0.000
	0.755	-0.055	0.012	0.000
	0.785	-0.046	0.011	0.000
	0.815	-0.045	0.013	0.000
	0.845	-0.039	0.013	0.004
	0.875	-0.034	0.014	0.012
	0.905	-0.031	0.015	0.047
	0.935	-0.035	0.018	0.050
	0.965	-0.022	0.027	0.416
	0.995	0.077	0.068	0.259
**FPI**	0.005	-0.504	0.056	0.000

	0.035	-0.546	0.054	0.000
	0.065	-0.501	0.044	0.000
	0.095	-0.496	0.044	0.000
	0.125	-0.457	0.036	0.000
	0.155	-0.436	0.038	0.000
	0.185	-0.443	0.036	0.000
	0.215	-0.412	0.028	0.000
	0.245	-0.393	0.023	0.000
	0.275	-0.395	0.029	0.000
	0.305	-0.385	0.026	0.000
	0.335	-0.371	0.024	0.000
	0.365	-0.341	0.017	0.000
	0.395	-0.319	0.012	0.000
	0.425	-0.294	0.015	0.000
	0.455	-0.279	0.014	0.000
	0.485	-0.262	0.012	0.000
	0.515	-0.239	0.010	0.000
	0.545	-0.229	0.011	0.000
	0.575	-0.214	0.012	0.000
	0.605	-0.205	0.013	0.000
	0.635	-0.206	0.014	0.000
	0.665	-0.210	0.013	0.000
	0.695	-0.192	0.013	0.000
	0.725	-0.190	0.013	0.000
	0.755	-0.191	0.014	0.000
	0.785	-0.197	0.014	0.000
	0.815	-0.190	0.015	0.000
	0.845	-0.203	0.016	0.000
	0.875	-0.213	0.017	0.000
	0.905	-0.245	0.022	0.000
	0.935	-0.321	0.024	0.000
	0.965	-0.425	0.037	0.000
	0.995	-0.507	0.053	0.000
**OD ovality**	0.005	-0.262	0.064	0.000
	0.035	-0.155	0.044	0.000
	0.065	-0.141	0.044	0.001
	0.095	-0.142	0.028	0.000
	0.125	-0.147	0.031	0.000
	0.155	-0.122	0.035	0.001
	0.185	-0.111	0.032	0.001
	0.215	-0.109	0.025	0.000
	0.245	-0.094	0.030	0.001
	0.275	-0.073	0.028	0.009
	0.305	-0.071	0.022	0.002
	0.335	-0.077	0.021	0.000
	0.365	-0.072	0.019	0.000
	0.395	-0.062	0.016	0.000
	0.425	-0.054	0.014	0.000
	0.455	-0.052	0.013	0.000
	0.485	-0.054	0.002	0.000
	0.515	-0.050	0.011	0.000
	0.545	-0.049	0.011	0.000
	0.575	-0.040	0.012	0.001
	0.605	-0.039	0.010	0.000
	0.635	-0.039	0.008	0.000
	0.665	-0.040	0.006	0.000
	0.695	-0.041	0.009	0.000
	0.725	-0.042	0.009	0.000
	0.755	-0.045	0.012	0.000
	0.785	-0.041	0.012	0.001
	0.815	-0.037	0.015	0.015
	0.845	-0.023	0.016	0.154
	0.875	-0.007	0.017	0.663

	0.905	0.014	0.021	0.519
	0.935	0.016	0.022	0.470
	0.965	0.051	0.037	0.166
	0.995	0.115	0.086	0.181
**OD area**	0.005	-0.647	0.041	0.000
	0.035	-0.569	0.048	0.000
	0.065	-0.468	0.045	0.000
	0.095	-0.408	0.040	0.000
	0.125	-0.415	0.032	0.000
	0.155	-0.415	0.034	0.000
	0.185	-0.413	0.033	0.000
	0.215	-0.367	0.028	0.000
	0.245	-0.340	0.027	0.000
	0.275	-0.323	0.026	0.000
	0.305	-0.308	0.021	0.000
	0.335	-0.282	0.022	0.000
	0.365	-0.261	0.020	0.000
	0.395	-0.228	0.017	0.000
	0.425	-0.193	0.016	0.000
	0.455	-0.174	0.012	0.000
	0.485	-0.168	0.010	0.000
	0.515	-0.157	0.010	0.000
	0.545	-0.151	0.011	0.000
	0.575	-0.140	0.012	0.000
	0.605	-0.137	0.011	0.000
	0.635	-0.132	0.011	0.000
	0.665	-0.124	0.011	0.000
	0.695	-0.124	0.011	0.000
	0.725	-0.133	0.010	0.000
	0.755	-0.139	0.010	0.000
	0.785	-0.153	0.009	0.000
	0.815	-0.159	0.012	0.000
	0.845	-0.174	0.013	0.000
	0.875	-0.184	0.011	0.000
	0.905	-0.216	0.017	0.000
	0.935	-0.251	0.014	0.000
	0.965	-0.352	0.023	0.000
	0.995	-0.779	0.057	0.000
**CRAE**	0.005	-0.138	0.061	0.023
	0.035	-0.281	0.050	0.000
	0.065	-0.261	0.046	0.000
	0.095	-0.247	0.044	0.000
	0.125	-0.218	0.036	0.000
	0.155	-0.197	0.037	0.000
	0.185	-0.191	0.035	0.000
	0.215	-0.159	0.028	0.000
	0.245	-0.141	0.027	0.000
	0.275	-0.116	0.027	0.000
	0.305	-0.116	0.025	0.000
	0.335	-0.106	0.023	0.000
	0.365	-0.093	0.021	0.000
	0.395	-0.083	0.016	0.000
	0.425	-0.083	0.012	0.000
	0.455	-0.081	0.011	0.000
	0.485	-0.068	0.010	0.000
	0.515	-0.062	0.009	0.000
	0.545	-0.060	0.011	0.000
	0.575	-0.063	0.012	0.000
	0.605	-0.056	0.012	0.000
	0.635	-0.054	0.012	0.000
	0.665	-0.055	0.012	0.000
	0.695	-0.049	0.013	0.000
	0.725	-0.047	0.012	0.000

	0.755	-0.042	0.012	0.001
	0.785	-0.044	0.013	0.000
	0.815	-0.040	0.014	0.004
	0.845	-0.053	0.015	0.000
	0.875	-0.053	0.015	0.000
	0.905	-0.069	0.018	0.000
	0.935	-0.071	0.018	0.000
	0.965	-0.068	0.031	0.026
	0.995	-0.216	0.079	0.006
**CRVE**	0.005	-0.729	0.059	0.000
	0.035	-0.698	0.047	0.000
	0.065	-0.701	0.047	0.000
	0.095	-0.648	0.045	0.000
	0.125	-0.613	0.036	0.000
	0.155	-0.571	0.038	0.000
	0.185	-0.537	0.035	0.000
	0.215	-0.542	0.029	0.000
	0.245	-0.517	0.027	0.000
	0.275	-0.482	0.028	0.000
	0.305	-0.442	0.025	0.000
	0.335	-0.418	0.023	0.000
	0.365	-0.388	0.020	0.000
	0.395	-0.339	0.016	0.000
	0.425	-0.299	0.014	0.000
	0.455	-0.262	0.014	0.000
	0.485	-0.227	0.010	0.000
	0.515	-0.199	0.012	0.000
	0.545	-0.177	0.011	0.000
	0.575	-0.163	0.012	0.000
	0.605	-0.152	0.012	0.000
	0.635	-0.140	0.013	0.000
	0.665	-0.129	0.013	0.000
	0.695	-0.126	0.013	0.000
	0.725	-0.122	0.012	0.000
	0.755	-0.128	0.012	0.000
	0.785	-0.120	0.012	0.000
	0.815	-0.127	0.014	0.000
	0.845	-0.112	0.015	0.000
	0.875	-0.111	0.015	0.000
	0.905	-0.116	0.019	0.000
	0.935	-0.145	0.016	0.000
	0.965	-0.187	0.030	0.000
	0.995	-0.148	0.066	0.026
**Vessel tortuosity**	0.005	0.254	0.058	0.000
	0.035	0.213	0.044	0.000
	0.065	0.168	0.039	0.000
	0.095	0.159	0.038	0.000
	0.125	0.166	0.031	0.000
	0.155	0.182	0.032	0.000
	0.185	0.177	0.030	0.000
	0.215	0.168	0.024	0.000
	0.245	0.172	0.023	0.000
	0.275	0.155	0.024	0.000
	0.305	0.139	0.022	0.000
	0.335	0.132	0.020	0.000
	0.365	0.124	0.017	0.000
	0.395	0.117	0.014	0.000
	0.425	0.113	0.011	0.000
	0.455	0.094	0.012	0.000
	0.485	0.086	0.009	0.000
	0.515	0.080	0.009	0.000
	0.545	0.067	0.010	0.000
	0.575	0.070	0.011	0.000

	0.605	0.065	0.011	0.000
	0.635	0.061	0.011	0.000
	0.665	0.062	0.011	0.000
	0.695	0.055	0.012	0.000
	0.725	0.061	0.011	0.000
	0.755	0.061	0.011	0.000
	0.785	0.066	0.012	0.000
	0.815	0.066	0.013	0.000
	0.845	0.078	0.014	0.000
	0.875	0.072	0.014	0.000
	0.905	0.092	0.018	0.000
	0.935	0.087	0.018	0.000
	0.965	0.132	0.028	0.000
	0.995	0.212	0.062	0.001
**Vessel FD**	0.005	1.704	0.071	0.000
	0.035	1.572	0.055	0.000
	0.065	1.434	0.048	0.000
	0.095	1.385	0.049	0.000
	0.125	1.333	0.041	0.000
	0.155	1.272	0.043	0.000
	0.185	1.211	0.044	0.000
	0.215	1.129	0.037	0.000
	0.245	1.060	0.035	0.000
	0.275	0.988	0.035	0.000
	0.305	0.913	0.032	0.000
	0.335	0.825	0.032	0.000
	0.365	0.763	0.029	0.000
	0.395	0.678	0.025	0.000
	0.425	0.595	0.024	0.000
	0.455	0.522	0.022	0.000
	0.485	0.458	0.016	0.000
	0.515	0.401	0.018	0.000
	0.545	0.350	0.016	0.000
	0.575	0.318	0.017	0.000
	0.605	0.291	0.017	0.000
	0.635	0.261	0.017	0.000
	0.665	0.233	0.017	0.000
	0.695	0.204	0.017	0.000
	0.725	0.187	0.015	0.000
	0.755	0.179	0.015	0.000
	0.785	0.157	0.016	0.000
	0.815	0.133	0.017	0.000
	0.845	0.125	0.017	0.000
	0.875	0.114	0.016	0.000
	0.905	0.112	0.022	0.000
	0.935	0.084	0.022	0.000
	0.965	0.077	0.031	0.012
	0.995	0.059	0.083	0.476
**Artery concavity**	0.005	-0.312	0.058	0.000
	0.035	-0.367	0.048	0.000
	0.065	-0.403	0.046	0.000
	0.095	-0.372	0.039	0.000
	0.125	-0.374	0.015	0.000
	0.155	-0.388	0.033	0.000
	0.185	-0.390	0.032	0.000
	0.215	-0.396	0.029	0.000
	0.245	-0.359	0.029	0.000
	0.275	-0.345	0.027	0.000
	0.305	-0.332	0.027	0.000
	0.335	-0.313	0.025	0.000
	0.365	-0.283	0.023	0.000
	0.395	-0.255	0.019	0.000
	0.425	-0.242	0.016	0.000

	0.455	-0.219	0.015	0.000
	0.485	-0.195	0.014	0.000
	0.515	-0.162	0.013	0.000
	0.545	-0.152	0.012	0.000
	0.575	-0.139	0.013	0.000
	0.605	-0.130	0.014	0.000
	0.635	-0.117	0.013	0.000
	0.665	-0.118	0.012	0.000
	0.695	-0.112	0.013	0.000
	0.725	-0.106	0.013	0.000
	0.755	-0.095	0.013	0.000
	0.785	-0.088	0.013	0.000
	0.815	-0.085	0.014	0.000
	0.845	-0.078	0.015	0.000
	0.875	-0.073	0.013	0.000
	0.905	-0.072	0.021	0.001
	0.935	-0.072	0.006	0.000
	0.965	-0.101	0.025	0.000
	0.995	-0.248	0.059	0.000
**Vein concavity**	0.005	-0.329	0.048	0.000
	0.035	-0.405	0.050	0.000
	0.065	-0.348	0.038	0.000
	0.095	-0.356	0.036	0.000
	0.125	-0.346	0.031	0.000
	0.155	-0.329	0.033	0.000
	0.185	-0.316	0.031	0.000
	0.215	-0.308	0.027	0.000
	0.245	-0.293	0.024	0.000
	0.275	-0.281	0.024	0.000
	0.305	-0.270	0.022	0.000
	0.335	-0.259	0.021	0.000
	0.365	-0.239	0.019	0.000
	0.395	-0.223	0.016	0.000
	0.425	-0.199	0.013	0.000
	0.455	-0.183	0.012	0.000
	0.485	-0.165	0.011	0.000
	0.515	-0.146	0.011	0.000
	0.545	-0.135	0.010	0.000
	0.575	-0.121	0.011	0.000
	0.605	-0.116	0.011	0.000
	0.635	-0.110	0.011	0.000
	0.665	-0.095	0.011	0.000
	0.695	-0.096	0.011	0.000
	0.725	-0.088	0.011	0.000
	0.755	-0.081	0.011	0.000
	0.785	-0.083	0.010	0.000
	0.815	-0.096	0.011	0.000
	0.845	-0.098	0.011	0.000
	0.875	-0.098	0.014	0.000
	0.905	-0.085	0.016	0.000
	0.935	-0.120	0.014	0.000
	0.965	-0.155	0.021	0.000
	0.995	-0.233	0.065	0.000

S6: Quantile regression* results for arteriovenous ratio (AVR): coefficient plot showing standardised beta vs refractive quantile (high hyperopia to high myopia from right to left)

**Model controlled for age, sex, corneal radius of curvature, optic disc-fovea distance, optic disc-fovea angle, optic disc orientation, foveal pixel intensity, optic disc ovality, optic disc area, vessel tortuosity, vessel fractal dimension, papillomacular arterial concavity, papillomacular venous concavity. AVR was the ratio of central retinal arteriolar equivalent to central retinal venous equivalent, both of which were corrected for ocular magnification. Other dimensional metrics included in the model were also corrected for ocular magnification.*

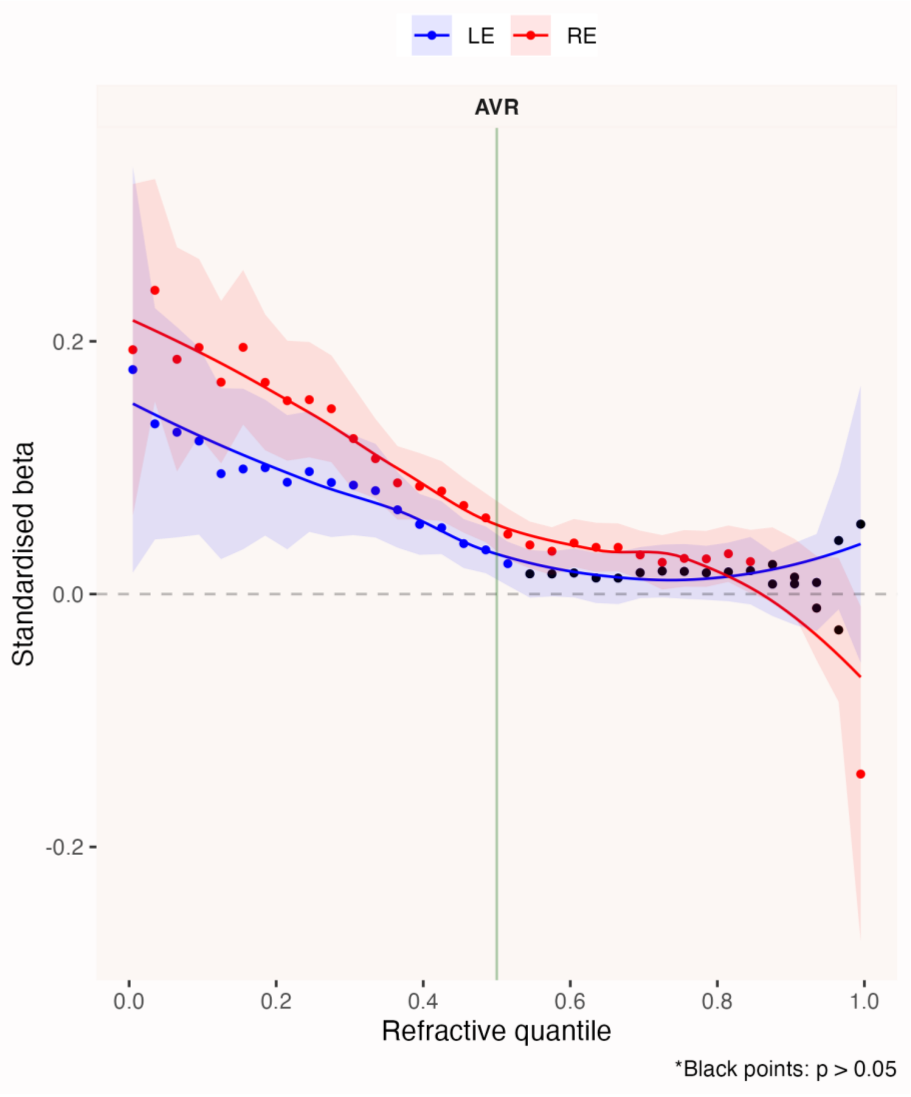

The positive beta coefficients indicated that AVR decreased as spherical equivalent refraction decreased (i.e., more negative).

S7: Quantile regression* results for central retinal arteriolar equivalent (CRAE) and central retinal venular equivalent (CRVE) *without* accounting for the effect of ocular magnification: coefficient plot showing standardised beta vs refractive quantile (high hyperopia to high myopia from right to left in each subplot)

Model controlled for age, sex, corneal radius of curvature, optic disc-fovea distance, optic disc-fovea angle, optic disc orientation, foveal pixel intensity, optic disc ovality, optic disc area, vessel tortuosity, vessel fractal dimension, papillomacular arterial concavity, papillomacular venous concavity, CRAE (for CRVE) and CRVE (for CRAE). All dimensional metrics* ***apart from* *CRAE and CRVE were corrected for ocular magnification.*

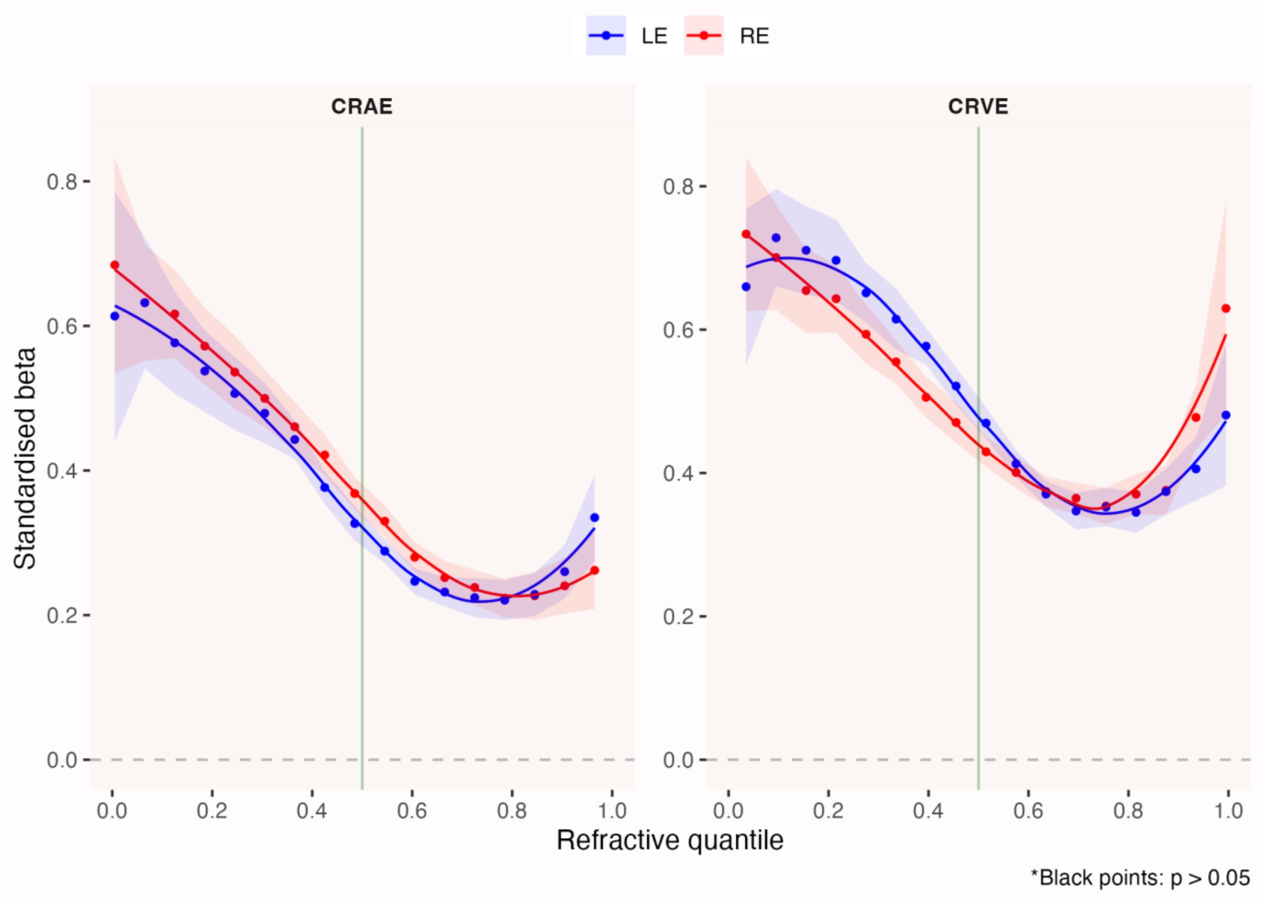

The positive beta coefficients across refractive error indicated that the central retinal arterioles and venules became narrower as spherical equivalent refraction decreased (i.e., more negative) when ocular magnification was **not** accounted for.
